# Nitrogen palaeo-isoscapes: Changing spatial gradients of faunal δ^15^N in late Pleistocene and early Holocene Europe

**DOI:** 10.1371/journal.pone.0268607

**Published:** 2023-02-06

**Authors:** Hazel Reade, Jennifer A. Tripp, Delphine Frémondeau, Kerry L. Sayle, Thomas F. G. Higham, Martin Street, Rhiannon E. Stevens

**Affiliations:** 1 UCL Institute of Archaeology, University College London, London, United Kingdom; 2 Scottish Universities Environmental Research Centre, Glasgow, United Kingdom; 3 Research Laboratory for Archaeology and the History of Art, University of Oxford, Oxford, United Kingdom; 4 Römisch-Germanisches Zentralmuseum, Forschungsinstitut für Archäologie Kompetenzbereich Pleistozäne und Frühholozäne Archäologie, Neuwied, Germany; University of Padova: Universita degli Studi di Padova, ITALY

## Abstract

Nitrogen isotope ratio analysis (δ^15^N) of animal tissue is widely used in archaeology and palaeoecology to investigate diet and ecological niche. Data interpretations require an understanding of nitrogen isotope compositions at the base of the food web (baseline δ^15^N). Significant variation in animal δ^15^N has been recognised at various spatiotemporal scales and related to changes both in baseline δ^15^N, linked to environmental and climatic influence on the terrestrial nitrogen cycle, and animal ecology. Isoscapes (models of isotope spatial variation) have proved a useful tool for investigating spatial variability in biogeochemical cycles in present-day marine and terrestrial ecosystems, but so far, their application to palaeo-data has been more limited. Here, we present time-sliced nitrogen isoscapes for late Pleistocene and early Holocene Europe (c. 50,000 to 10,000 years BP) using herbivore collagen δ^15^N data. This period covers the Last Glacial-Interglacial Transition, during which significant variation in the terrestrial nitrogen cycle occurred. We use generalized linear mixed modelling approaches for interpolation and test models which both include and exclude climate covariate data. Our results show clear changes in spatial gradients of δ^15^N through time. Prediction of the lowest faunal δ^15^N values in northern latitudes after, rather than during, the Last Glacial Maximum is consistent with the Late Glacial Nitrogen Excursion (LGNE). We find that including climatic covariate data does not significantly improve model performance. These findings have implications for investigating the drivers of the LGNE, which has been linked to increased landscape moisture and permafrost thaw, and for understanding changing isotopic baselines, which are fundamental for studies investigating diets, niche partitioning, and migration of higher trophic level animals.

## 1. Introduction

Nitrogen isotope ratio analysis (expressed as δ^15^N) of biological tissue is frequently used in archaeology and palaeoecology to investigate dietary behaviours, ecological niche, and past food webs [1–3]. Specifically, δ^15^N is used to infer information about trophic structures. Obtaining reliable estimations of faunal trophic position requires understanding of the isotope compositions at the base of the food web. In other words, knowledge of the plant and soil δ^15^N values upon which the fauna lived and fed (hereafter termed baseline δ^15^N). However, this information is not usually readily obtainable from archaeological or palaeontological contexts, where the preservation of plant and/or soil material suitable for analysis can be limited. Moreover, plant and soil δ^15^N is highly heterogeneous and is not static in space or time, complicating inferences of baseline δ^15^N available to fauna.

Many interconnected factors exert influence on plant and soil δ^15^N and nitrogen cycling in the terrestrial environment [4]. These relate to climate, plant functional type, mycorrhizal associations, soil characteristics, and the availability of different forms of nitrogen [5–8]. On global and continental scales strong, albeit indirect, relationships exist between plant δ^15^N and temperature and precipitation [6, 7]. These relationships are also expressed over smaller spatial scales with strong altitudinal gradients [9, 10]. Likewise, such spatial relationships are also represented in faunal δ^15^N values [9, 11, 12]. However, differences in dietary and mobility behaviours between different species, populations, and individuals introduce additional variations into the faunal δ^15^N signal [1, 3]. Indeed, while δ^15^N analysis of biological tissues is frequently used in archaeology and palaeoecology to investigate dietary behaviours and ecological niche, our ability to decipher environmental influence from feeding behaviour remains an ongoing challenge.

On long timescales (10^3^ to 10^5^ years) significant temporal variation has been identified in herbivore δ^15^N [13–25]. This variation has been interpreted as representing changes to baseline δ^15^N in response to climatic and environmental drivers. Most notably, a large decrease and then rapid increase in herbivore δ^15^N occurred during the Late Glacial, between approximately 17,000- and 12,000-years before present (BP) [15–17, 20–23, 25, 26]. This trend occurs in multiple species, across a wide range of mid and high latitude environments and in recent years has been termed the Late Glacial Nitrogen Excursion (LGNE) [25]. As the body of late Pleistocene herbivore δ^15^N data has grown, spatial and temporal asynchronicities in the LGNE are becoming increasingly apparent [3, 25]. Similarly, significant differences in species-specific δ^15^N variation are also recognised [1, 3].

Through this increasing body of data, significant new opportunities to investigate spatiotemporal patterns in herbivore δ^15^N and the underlying controls of this variation are emerging. Isoscape approaches (modelling of isotope spatial variation) have proved useful tools for investigating isotopic spatial variability in present-day marine and terrestrial ecosystems but are yet to be widely and routinely applied to palaeo-focused research [27–30]. Here, we create time-sliced isoscape prediction maps of herbivore collagen δ^15^N through the late Pleistocene and early Holocene periods in Europe. Time-sliced spatial interpolation offers the potential to assess changing spatial gradients of δ^15^N through time. Combining this analysis with high resolution climate model data [31] opens up a significant new avenue of research through which the potential drivers of the LGNE can be investigated. Improved characterisation of spatiotemporal trends in herbivore δ^15^N may also ultimately contribute to more robust trophic structure analysis of archaeological and palaeontological materials. This is particularly important as many palaeo-focused studies use herbivore δ^15^N, in the absence of suitable plant samples, to infer baseline δ^15^N values for terrestrial food web analysis and in the interpretation of data from higher trophic level animals in relation to mobility, migration, and dietary research.

## 2 Materials and methods

### 2.1 Data compilation

Newly generated and previously published ungulate herbivore collagen δ^15^N from late Pleistocene and early Holocene European contexts were compiled for latitudes between 35°N and 60°N and longitudes between 10°W and 30°E. Temporal scope was restricted to before the 8.2 ka BP climatic event [32], to avoid capturing human-influence on baseline δ^15^N that occurred through agricultural developments with the onset of the Neolithic [33, 34], and after 50 ka BP, which is nearing the current limit of radiocarbon dating and calibration [35]. Data come from both archaeological and palaeontological assemblages. Latitude and longitude were established for each sample based on published information. For most samples, a latitude and longitude was either directly given for that sample in the publication of the δ^15^N data, or for the site from which the sample came. In some instances, latitude and longitude were estimated based on a publication’s description of the site location/map given in that publication. We believe the resultant compilation (n = 3,733) captures the majority of available ungulate δ^15^N data from the time period and geographical region of focus, enabling major spatial and temporal trends in δ^15^N to be evaluated.

In screening the data we exclude δ^15^N with an associated C/N atomic ratio <2.9 or >3.6, where a C/N atomic ratio was not published, or where the data publication indicated the result was unreliable (n = 91). We exclude data where taxonomic identification was uncertain (n = 43) or where samples came from juvenile animals (n = 14). All antler (n = 110) and tooth (n = 46) samples from Cervid species (*Alces alces*, *Capreolus capreolus*, *Cervus elaphus*, *Megaloceros giganticus*, *Rangifer tarandus*, and *Rupicapra rupicapra*) were excluded to limit seasonal biasing in the data [36–39]. Finally, any data which were identified as duplicate analyses on the same sample/individual animal were omitted (n = 336).

Compiled data were divided into 7 temporal bins (Table 1); to minimise the potential of averaging data across different climatic states/environmental conditions, whilst not overly limiting the number of data included in each time bin, we base our time bins on known major climatic events [40, 41]. We recognise that further climate events occurred within our selected bins, and that their expression is asynchronous across the region of study, but without a greater sample size and/or improvements in the accuracy to which sample age can be estimated, analysis at greater temporal resolution is not possible. In particular, the earliest of our two bins (early Oxygen Isotope Stage 3 (EOIS3) and late Oxygen Isotope Stage 3 (LOIS3)) capture data from multiple different climate states. For directly radiocarbon dated samples, calibration was performed using OxCal (version 4.4) [42] and the IntCal20 calibration curve [35]. Dates were binned based on the median of the 95.4% probability calibrated age range. For samples where age is based on stratigraphic provenance (context dated samples), time bin assignment was based on the age of the assemblage given in the original publication of the data, or most recent age model for the site in cases where the chronological position of an assemblage had been subsequently revised. For data where a secure age assignment could not be made, or when an age assignment spanned the boundary between two temporal bins, the data were excluded (n = 375). The resultant screened dataset contained 2,718 *δ*^15^N values, as reported in the Supporting Information (S1 Dataset). In total the data include 717 previously unpublished δ^15^N values (n = 470 in the screened data set) and 206 previously unpublished radiocarbon dates (n = 197 in the screened data set). Methods of sample preparation and analysis for the newly generated isotope data and radiocarbon determinations are provided in the Supporting Information (Section 1 in S1 File).

**Table 1:** Time bins and climate model time steps used in this study.

Time bin (abbreviation)	Upper limit (year cal BP)	Lower limit (year cal BP)	Climate model time step
Early Holocene (EH)	11,650	8,190	11,000
Younger Dryas (YD)	12,850	11,650	12,000
Late Glacial Interstadial (LGI)	14,650	12,850	14,000
Last Glacial Termination (LGT)	19,500	14,650	15,000
Last Glacial Maximum (LGM)	27,500	19,500	24,000
Late Oxygen Isotope Stage 3 (LOIS3)	39,850	27,500	36,000
Early Oxygen Isotope Stage 3 (EOIS3)	50,000	39,850	42,000

Elevation and bioclimatic covariate data were assembled for each δ^15^N sample, based on its geographic origin and time bin assignment. Elevation data was extracted from the Global Multi-Resolution Terrain Elevation Data 2010 (GMTED2010) model [43]. As this elevation data is relative to present day sea level, it does not account for temporal changes in sea level or isostatic changes related to the growth and melting of ice sheets. Bioclimatic data was extracted from 0.5° resolution, biased-corrected combined HadCM3 and HadAM3H time series climate simulations [31]. Data for these variables is available at a temporal resolution of 1,000-years for 21,000 years BP to present and at 2,000-year intervals prior 21,000 years BP. The distribution of samples within each time bin was evaluated using 1000-year bins and the modelled time step most closely corresponding to the greatest prevalence of data within the upper and lower limit of the event boundary was selected (Table 1).

### 2.2 Data exploration and analysis

All statistical analyses were performed using the R programming language (version 4.0.4) [44]; the R script is provided in the Supporting Information (S2 Dataset). The compiled data were first evaluated for potential species-based effects on δ^15^N related to diet, habitat preference, and ecology. While differences were identified between species, these were unsystematic, varying by location and time period (full analysis is reported in Section 2 in S1 File). As such, no species-based data normalisation procedures were applied prior to geostatistical analysis, although we also consider scope for species-specific analysis where sample size permits in Discussion Section 4.2.

Spatial structures in the data were evaluated by time bin using Anselin’s Local Moran’s I and Global Moran’s I tests [45, 46]. Coincident sample points were spatially jittered around 0.1° latitude and longitude. Spatial relationships were defined using inverse Euclidean distance. Row standardization was applied to the spatial weights to account for unequal sample distribution and corrections based on the False Discovery Rate were applied to cluster and outlier p-values to account for spatial dependency. The calculated local Moran’s I index (Ii), z-score, and adjusted p-value we used to determine clusters and outliers, where a positive Ii value indicated a sample is surrounded by other samples with similar values, and a negative Ii value indicated a sample is surround by other samples with dissimilar values. Clusters were defined as those where positive Ii values occur above a significance threshold of 0.95, and outliers as those where negative Ii values occur above a significance threshold of 0.95.

Geostatistical analysis followed a generalised linear mixed-effects model approach (GLMM), as described by [47, 48] using the R package SpaMM [49]. We first aggregated our data by sample location, calculating the mean, variance, and number of δ^15^N observations per site and time bin. We then fitted the isocape model in a two-step process utilising a residual dispersion and mean model. Our residual dispersion model is a Gamma GLMM fitted to the calculated variance using the restricted maximum likelihood method [47]. We included a Matérn correlation function as a spatially-structured random effect to account for spatial autocorrelation, and sample location (Site) as an uncorrelated random effect. Using the fit of the residual dispersion model (fitted using the fitme function in SpaMM [49]) we then predicted a residual error for each sample location model (using the predict function in SpaMM [49]). This residual error was included in our linear mixed-effects (LMM) mean model, which also incorporated fixed and random effects [47]. Various mean models fitted to site mean δ^15^N were tested (S5.1 Table in S1 File), with different bioclimatic covariate data included as fixed effects, and the Matérn correlation function and sample location as random effects. Combinations of fixed effects were selected for inclusion in the models based on known empirical relationships between δ^15^N and environmental variables, and on interrogation of correlations between faunal δ^15^N and modelled climatic data, tested with Pearson’s correlation analysis (Section 4 in S1 File). Model performance was evaluated using the conditional Akaike Information Criterion (cAIC) [49], and the best performing models were then used to predict δ^15^N across our study area and visualise these results as time binned δ^15^N isoscapes (interpolated prediction and variance surfaces). A workflow for our methodology is reported in Section 5 of S1 File (S5.1 Fig in S1 File) and full details of the modelling approach is available in Courtiol and Rousset [47].

## 3 Results

### 3.1 Data summary and spatial structure

**Fig 1:**
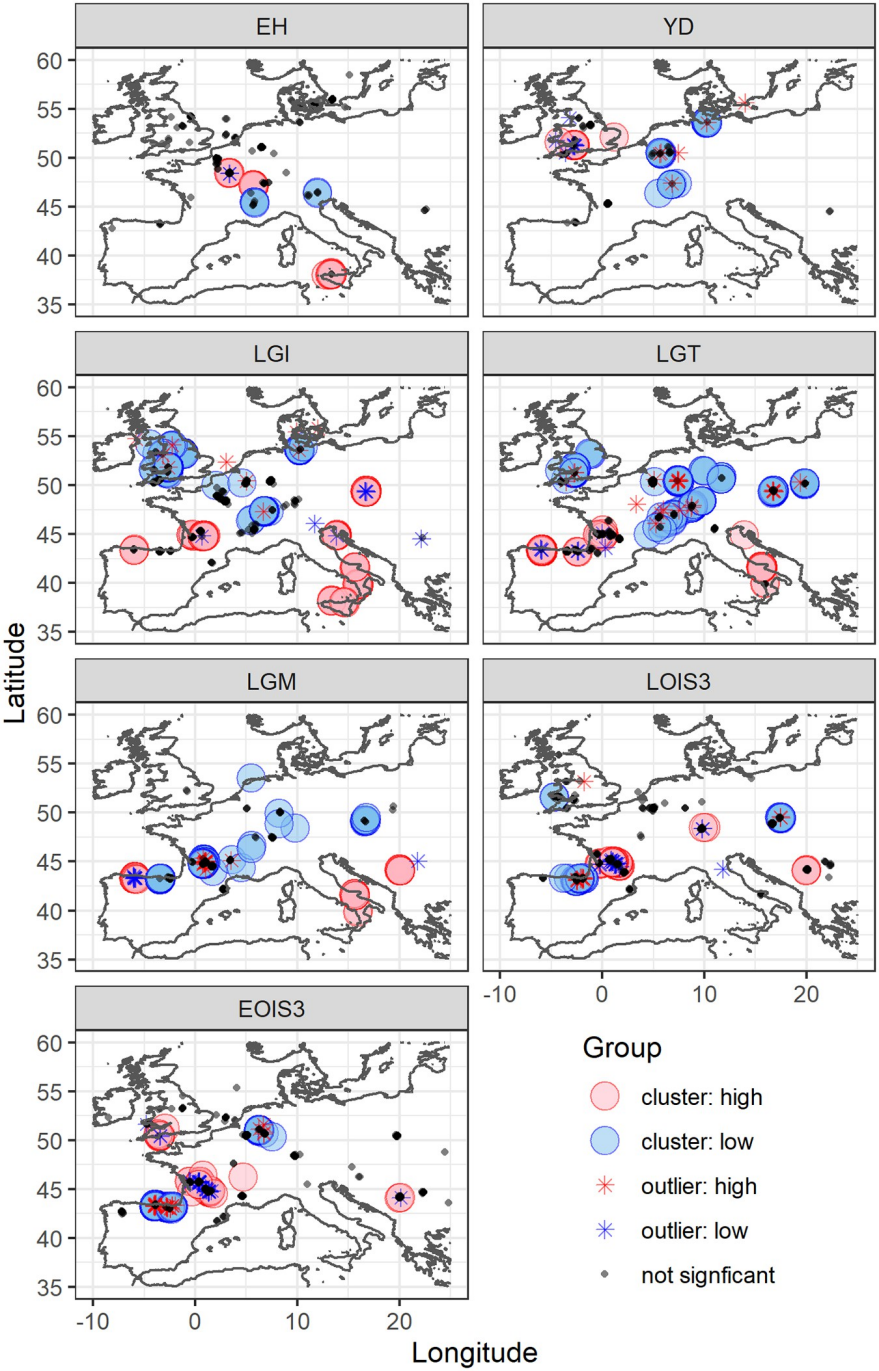
Visualisation of cluster (circles) and outlier (stars) analysis. High δ^15^N values are indicated in red, low δ^15^N values are indicated in blue. Coastline map from [50].

Within the assembled data (n = 2,718) mean δ^15^N is 4.1 ± 2.0‰, ranging from -0.9‰ to 11.9‰ (S3.1 Table, S3.1 Fig in S1 File). Cluster and outlier analysis (Anselin’s Local Moran) highlight underlying spatial trends in the data (Fig 1, S3.2 Table in S1 File), with the pattern of spatial clustering varying by time bin. Specifically, the analysis shows a north-south divide in clustering of low and high δ^15^N values respectively, for the LGM, LGT, and LGI time bins. The approximate latitude of the boundary between low δ^15^N and high δ^15^N clusters is at its most southerly extent during the LGM (Fig 1). The number of spatial outliers also varies by time bin (S3.2 Table in S1 File). These spatial outliers could indicate incorrect time bin assignment based on uncertainties in age estimation or could represent true local variability in the faunal δ^15^N data produced by localised environmental variation and/or differences in animal ecology. The outliers may also be an artifact of our data aggregation procedure, particularly for the EOIS3 and LOIS3 time bins which span long periods; it is entirely possible that some samples in close geographical proximity to one another are temporally disparate, and thus combine data representing differing climatic/environmental states (Section 3 in S1 File for more detailed discussion). As our interest is in investigating generalised continental-scale spatial patterns, the decision was taken to omit these outliers (n = 186) from further analysis. All time bins displayed significant spatial autocorrelation (Global Moran I), indicating systematic spatial variation in the data (S3.2 Table in S1 File). The strength of this spatial relationship varied in time, being strongest for the Last Glacial Maximum (LGM), Last Glacial Termination (LGT), Late Glacial Interstadial (LGI) and Younger Dryas (YD) time bins, and weaker for EOIS3, LOIS3, and early Holocene (EH) time bins.

### 3.2 Geostatistical analyses: Isoscapes

**Fig 2:**
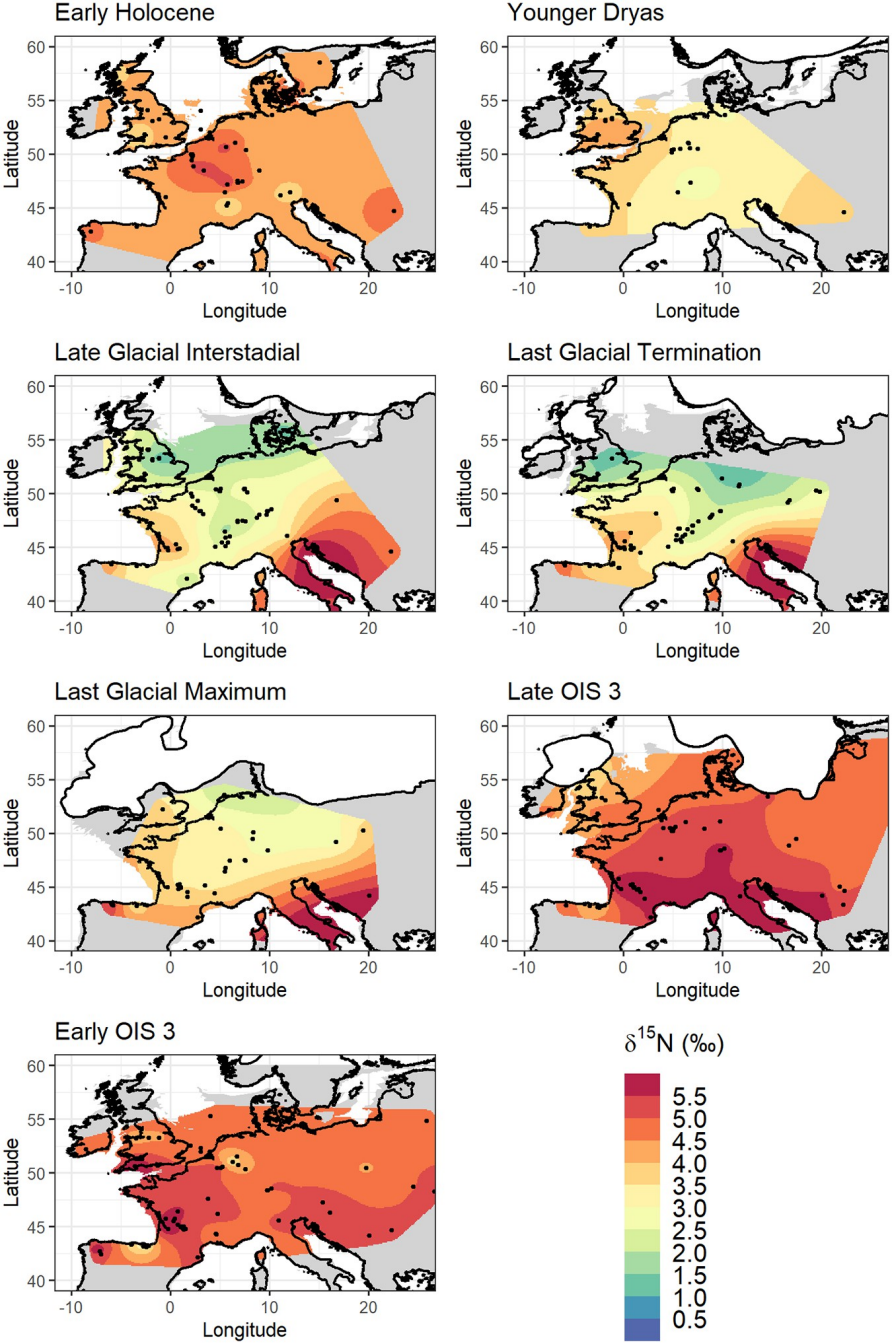
δ^15^N isoscape prediction surfaces, modelled using random effects only. Palaeocoastline data from [65], ice sheet extent from [66] and modern coastline from [50].

**Fig 3:**
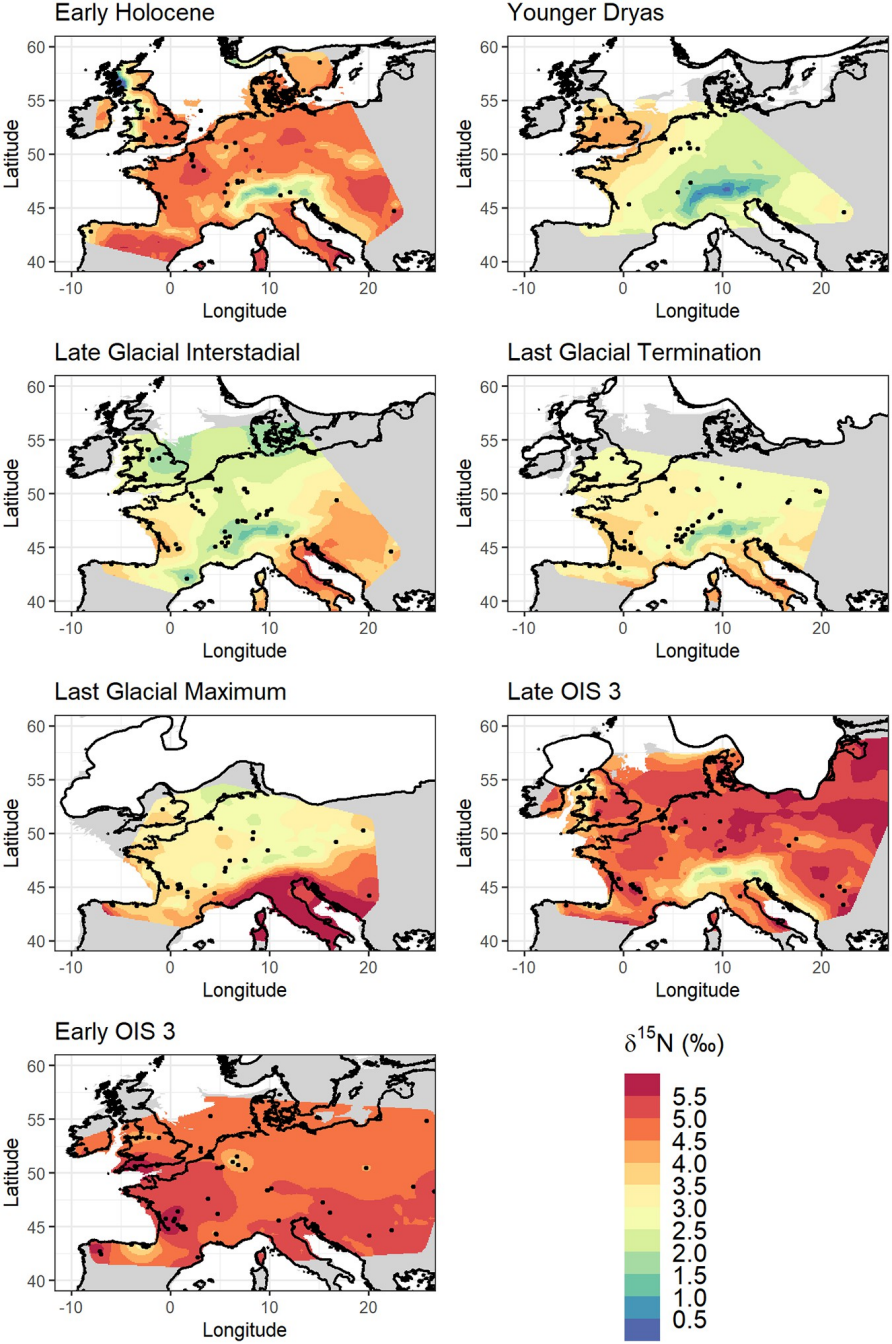
δ^15^N isoscape prediction surfaces, best performing model incorporating climatic fixed effect(s) for each time bin. Palaeocoastline data from [51], ice sheet extent from [52] and modern coastline from [50].

Spatial interpolation was investigated both with and without the inclusion of climate data as fixed effects (Table 2). In selecting which variables and combinations of variables to test as fixed effects in the isoscape models, we considered known empirical relationships between δ^15^N and climate in the modern environment, the strength of correlations between site-mean faunal δ^15^N and modelled terrestrial climatic variables, and issues of collinearity between available climatic variables (full analysis reported in Section 4 in S1 File). From this analysis the variables selected as fixed effects for model testing were mean annual temperature (MAT), mean annual precipitation (MAP), temperature of the warmest quarter (temp.warm), precipitation of the warmest quarter (precip.warm) and precipitation of the coldest quarter (precip.cold) (S4.2–S4.6 Figs in S1 File). Different combinations of these variables were considered, and model performance was evaluated using the cAIC (Table 2 and Section 5 in S1 File). Given that the strength of correlation with covariate data differed by time bin, it was unsurprising to find that the best fit model also differed by time bin. For all time bins, models including bioclimatic fixed effects performed better than the model where no fixed effects were included, with the exception of the LGI. However, for all time bins the changes in the cAIC criterion between different models were for the most part extremely small (Table 2), suggesting most models performed similarly well, and the inclusion of bioclimatic variables did not significantly improve model performance for any time bin. The resultant isoscape prediction surfaces, both without and with the inclusion of climatic data (Figs 2 and 3), show similar continental-scale spatial patterns of δ^15^N for most time bins, although with some notable differences. Importantly, the strength of expression of the north-south gradients in δ^15^N is more muted when climatic variables are incorporated, particularly for LOIS3, the LGT, and the LGI. Greater localised variation in δ^15^N is also apparent when climatic variables are incorporated, related to localised spatial climatic gradients, such as those that exist across areas of varying topography (e.g. the Alps mountain range). Prediction variance surfaces for the two outputs are similar (S5.2 and S5.3 Figs in S1 File), showing variance ranging from >1.5‰ across much of the prediction area for LOIS3 to <0.5‰ across much of the prediction area for LGI. Variance is lowest closest to sample locations, but otherwise does not appear to be spatially structured. Comparison of predicted δ^15^N values to observed δ^15^N values at sample locations also shows for the two outputs that the inclusion bioclimatic variables did not significantly improve model performance for any time bin (S5.4 and S5.5 in S1 File). Notably the comparison of predicted versus observed δ^15^N values appears to show a tendency for some models to systematically under predict high δ^15^N values and overpredict low δ^15^N values.

**Table 2:** Model fit results (cAIC) for faunal nitrogen isoscape prediction models.

Model parameters	cAIC						
	EH	YD	LGI	LGT	LGM	LOIS3	EOIS3
No fixed effects + spatial + site	213.3	77.9	***110*.*5***	132.6	54.1	170.2	181.2
MAT + spatial + site	212.7	78.0	110.8	***131*.*8***	54.2	170.8	***181*.*0***
MAP + spatial + site	210.9	77.9	110.9	132.9	54.3	***169*.*3***	181.9
MAT + MAP + spatial + site	***210*.*3***	78.1	111.2	132.4	54.3	169.3	181.1
MAT + MAP + MAT:MAP + spatial + site	210.9	78.3	111.6	132.5	54.8	169.7	182.0
temp.warm + spatial + site	211.9	***77*.*7***	110.7	131.9	56.6	170.4	181.8
precip.warm + spatial + site	211.4	77.9	110.8	133.1	54.0	169.4	181.6
precip.cold + spatial + site	211.9	78.0	111.1	132.9	53.7	169.9	182.2
temp.warm + precip.warm + spatial + site	210.8	77.8	111.1	132.2	56.4	169.8	182.2
temp.warm + precip.cold + spatial + site	211.2	78.0	111.2	132.1	55.6	170.0	182.7
Precip.cold + precip.warm + spatial + site	211.4	78.1	111.3	133.3	***52*.*9***	169.5	182.5
temp.warm + precip.cold + precip.warm + spatial + site	211.2	78.0	111.4	132.4	54.9	169.8	183.0

Fixed effects tested include mean annual temperature (MAT), mean annual precipitation (MAP), temperature of the warmest quarter (temp.warm), precipitation of the warmest quarter (precip.warm) and precipitation of the coldest quarter (precip.cold). A spatially-structured random effect following a Matérn correlation structure using latitude and longitude to compute distances between observations (spatial), and an uncorrelated random effect identical for all observations from the same location (site) were also included. Further fit results are given in Section 5 of S1 File. The best performing model, based on cAIC, is indicated in bold and italics.

## 4. Discussion

### 4.1 Evaluating spatial gradients and drivers of δ^15^N variability in the past

The results presented here provide the means to visualise the spatiotemporal character of changing faunal δ^15^N, and, when combined with the recent publication of high-resolution climate model data [31], interrogate potential links between faunal δ^15^N and climatic variables at a resolution not previously achievable.

The derived isoscapes (Fig 2) show the development of a north-south spatial gradient in faunal δ^15^N during LOIS3, which becomes gradually more pronounced through the LGM, LGT and LGI. This contrasts to EOIS3, YD, and EH where strong spatial gradients in δ^15^N are absent. The amplification of the latitudinal gradient reaches its maximum during the LGT and LGI, and notably appears to primarily be driven by a decrease in δ^15^N in northerly locations, rather than by an increase in δ^15^N in southerly locations. The fact that the lowest δ^15^N values occur during the LGI, a relatively warm climatic period, and not during the LGM, the coldest part of the last glacial cycle, show that temperature is not the primary driver of variation. We particularly draw attention to the location of the lowest predicted δ^15^N values, in regions that were either glaciated or were immediately proximal to the British, Scandinavian, and Alpine ice sheets during the LGM (Figs 2 and 3). It is noteworthy that δ^15^N values of <2‰ only occur within the zone of continuous permafrost that existed across Europe at the height of the last glacial [53], and their occurrence within this zone is only after the onset of deglaciation and thaw. The role of increased landscape moisture driven by increased precipitation and increased input of meltwater from icesheets and thawing permafrost has long been suggested as a driver of the LGNE [17, 18, 22, 23, 25], and the results presented here add further weight to this interpretation. This environmental change would have both altered the floral community in such landscapes and altered the form and source of nitrogen available to vegetation, with microbially-mediated changes in N cycling between pools of NO_3_^-^ (nitrate), NH_4_^+^ (ammonium), and N_2_ (elemental nitrogen) resulting in changing plant δ^15^N [54–56]. Biogeochemical cycles and microbial activity in cold environments may be particularly sensitive to changes in soil moisture content, O_2_ status, and temperature [54, 57, 58]. In this regard, the use of geostatistical interpolation to reconstruct changing δ^15^N spatiotemporal gradients may provide the means to further interrogate sub-continental scale processes of permafrost thaw and changing landscape moisture during the terminal Pleistocene in Europe. If increased data availability in coming years were to enable faunal isoscape mapping at an increased temporal resolution for the late glacial, it would certainly be of interest to compare these to contemporaneous maps of changes in the distribution of European permafrost.

That said, the relationship between the strength of spatial gradients in δ^15^N and the correlation between δ^15^N and climatic variables requires some consideration. Time bins with the strongest spatial gradients/spatial autocorrelation (LGM, LGT, LGI) also show the strongest correlation with climatic variables such as mean annual temperature, and temperature and precipitation of the warmest quarter (S4.2, S4.4, S4.5 Figs in S1 File). Conversely, time bins with absent (EOIS3, YD, EH) or weaker (LOIS3) spatial gradients show less correlation between δ^15^N and climatic variables. Taking mean annual temperature (S4.2 Fig in S1 File) as an example and considering the range of temperatures covered by the data for each time bin, there is no obvious relationship between this and the strength of the predicted spatial gradients. For example, the sample locations for the EH and LGI time bins cover a similar range in temperatures (c. 5 to 15°C), as do those for the LGM and EOIS3 (c. 0 to 10°C), but each display quite different spatial patterns. Likewise, the distribution of samples across our sampling area cannot explain all the derived patterns. While for EH and YD sample distribution and number is relatively poor compared to other time bins, this is not the case for EOIS3, which has a similar number and geographical distribution of samples as the LGT and LGI time bins. Indeed, for EOIS3 we suspect the most plausible explanation for the lack of a recognised spatial gradient is the previously mentioned effect of data aggregation across multiple climatic events. This may also go some way to explaining why there are a wide range of δ^15^N observed for very similar temperatures. However, this explanation is unsatisfactory for the early Holocene and Younger Dryas. A more plausible explanation for these time bins is that the reduced spatial gradient in δ^15^N is the result of less pronounced climatic gradients across the area of Europe our data covers, as is evidenced in both proxy-based data and model simulations [59, 60]. Additionally for the Younger Dryas, the expression of the rapid cooling event in European proxy archives is asynchronous [61, 62]; this, coupled with the brevity of the event (c. 1200 years), potential lag in environmental response, and uncertainty in assigning faunal samples to such a narrow age bracket may go some way to explaining difficulties in understanding the resultant isoscape model.

With these discussion points in mind, and despite our finding that the inclusion of climatic covariate data did not improve isoscape model performance (Table 2), our analysis nonetheless shows that for some time bins palaeo-fauna δ^15^N is correlated with modelled temperature and precipitation (S4.2-S4.6 Figs in S1 File). What is most striking about the investigated palaeo-fauna δ^15^N–climate correlations, is the strength of the relationship between site mean faunal δ^15^N and MAT (as well as temperature and precipitation of the warmest quarter) during the Last Glacial Maximum, Last Glacial Termination and Late Glacial Interstadial (S4.2; S4.4 and S4.5 Figs in S1 File). Such relationships are stronger than those observed for modern soil/plant δ^15^N –MAT relationships [6, 7]. Interestingly, while the relationship between foliar and soil δ^15^N and MAT has been shown not to hold true in modern low temperature environments (<-0.5°C for foliar δ^15^N and <9.8°C for soil δ^15^N [6, 7]), we do not observe such inflection points in our data, although it should be noted that few samples come from environments where MAT is predicted to be <0°C.

Also notable is the stronger correlation between site mean faunal δ^15^N and precipitation of the warmest month, compared to the relationship with MAP (S4.3 and S4.5 Figs in S1 File). The relatively weak correlation between faunal δ^15^N and MAP for most time bins does not mirror those seen in the modern environment. In part, this may relate to the comparatively more complex nature of reconstructing palaeo-precipitation, and the poorer performance of precipitation models when compared to proxy data-based reconstructions, than for reconstructing temperature [31]. However, this result may also represent the importance of the complex interplay of temperature and precipitation in determining δ^15^N. Further, the seasonal cycle of plant growth and N requirements/availability may be responsible for seasonally distinct relationships between δ^15^N and climate.

Keeping in mind the caveats of our analysis, that; 1) faunal δ^15^N is only indirectly related to climate, being mediated also by the interplay of species-specific characteristics and inter-species interactions, and by N-cycle dynamics; that 2) the climate data we are using is modelled output, not empirical measurements; and 3) the assembled data aggregates δ^15^N across multiple species and potentially disparate time periods, the presented results nonetheless offer an intriguing insight into the spatial and temporal variability of δ^15^N in the past.

### 4.2 Species-specific spatial gradients of δ^15^N variability

One of the primary challenges in understanding the variability present in fossil δ^15^N data is to distinguish between environmental effects and the effects of dietary and ecological differences between species, and the variability that can occur in both effects across space and time. Some previous studies of environmental change that utilise faunal δ^15^N restricted analyses to single species to limit variability introduced by dietary ecology (e.g. [17, 23]). Others take data from more than one species with similar dietary characteristics and apply data normalisation/transformation procedures (e.g. [25]). The former severely limits the size of the data set available for analysis, while the latter, when used to infer environmental change, relies on the assumption that species’ dietary behaviour and isotope niche relative to one another have remained stable through time. Empirical evidence suggests this assumption is problematic [1, 3, 63, 64]. Indeed, species most capable of dietary flexibility are often most successful at adapting to changes in local environmental conditions [65, 66], and thus it is these species that are most abundant in the fossil record and remain present across major climate transitions. In this study we applied no correction or data transformation procedure to account for species-based differences, and instead considered only site mean δ^15^N values in the models thus far presented. Our primary reasoning for this is that while species-based differences are present in the data, neither the option of restricting analysis to a single species or applying species-based corrections to a multiple-species analysis were appropriate in this instance (Section 2 in S1 File).

**Fig 4:**
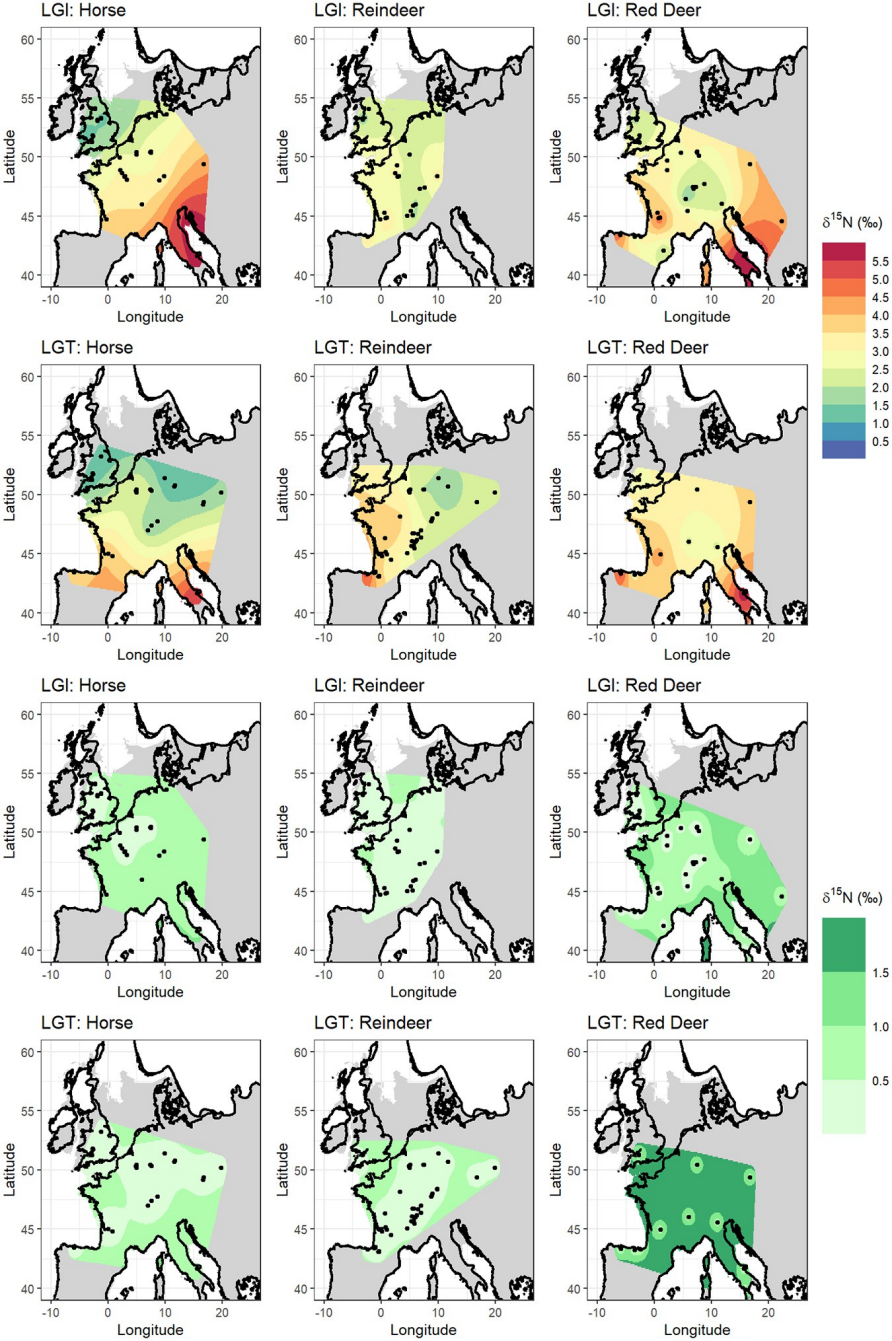
δ^15^N isoscape prediction and variance surfaces for *Equus* sp., *Rangifer tarandus*, and *Cervus elaphus* for the Last Glacial Termination and Late Glacial Interstadial time bins. Linear mixed models were run without the addition of environmental covariate data. Palaeocoastline data from [51], ice sheet extent from [52] and modern coastline from [50].

Nonetheless, it is important to consider the implications of our approach compared to model outputs when different species are considered independently. While the data is not of sufficient quantity to enable species-specific isoscapes to be constructed for all time bins and species, they can be considered in some contexts. Our data is dominated by 3 species: horse (25%), reindeer (24%) and red deer (30%), and while their geographical distribution varies considerably in time (S6.1 Fig in S1 File), data is of sufficient quantity and comparable geographic distribution to consider simple isoscape models for each of these species for the LGT and LGI time bins (Fig 4).

From this analysis horse can be seen to display the strongest spatial gradients in δ^15^N in both the LGT and LGI, with lowest values in the north and northwest of the interpolation area, and highest values in the south. North-south gradients in δ^15^N are also seen in the reindeer and red deer data but are more muted. While these differences likely stem from differences in dietary ecology and mobility (see Section 2 of S1 File for more detailed discussion), the implications for this in our objective of understanding large-scale changing spatial gradients in δ^15^N requires consideration. Deciphering the relative contributions of dietary behaviour and environmental influence on the δ^15^N signal is extremely challenging, and it may never be possible to fully disentangle the two by measuring bulk collagen δ^15^N alone; as localised environmental conditions exert strong influence on feeding behaviours and diet, the two are inextricably linked. In the context of late Pleistocene northern Europe, tooth meso- and microwear analysis confirm horse most likely had a graze-dominated diet, red deer a browse-dominated diet, and reindeer a mixed diet [66]. However, the extent to which these different plant types in the diet can be equated to isotopically distinct diets is debateable. As Schwartz-Narbonne et al. [3] discuss, while at the most generalised scale patterns of δ^15^N in tundra ecosystems can be summarised as shrub < lichen < herb < fungi, there are examples where this does not hold true in either space or time [67–70]. If this generalised plant type δ^15^N pattern was taken at face value and applied to the late Pleistocene European herbivores, a pattern of red deer < reindeer < horse would be expected; in fact, the opposite pattern is identified.

Part of the difficulty in relating faunal δ^15^N to plant δ^15^N is the issue of scale. Plant δ^15^N is highly heterogenous and is related to N availability and a plant’s ability to utilise and acquire different forms of N, which are influenced by root depth and mycorrhizal association, as well as environmental factors which can vary on a sub-annual scale [4]. In comparison, one collagen δ^15^N analysis represents a homogenised data point, averaging a multitude of plant δ^15^N values at a spatial scale equivalent to the animal’s home range (which can vary considerably between species) and a temporal scale of several years. A further consideration in interpreting the faunal signal, particularly that of reindeer, is the inclusion of lichen in the diet. Lichens fix nitrogen from the atmosphere, and therefore species consuming a significant proportion of lichen may display δ^15^N signatures decoupled from environmental-mediated changes in vegetation δ^15^N. However, the amount of lichen consumed, and its contribution to the reindeer bone collagen δ^15^N signal cannot be easily discerned. For example, significant differences in the amount of lichen incorporated into reindeer diets between the LGI and YD in northern Europe, based on tooth meso- and micro-wear analysis, did not translate to differences in bone collagen δ^15^N [71]. As such, we decided that the exclusion of reindeer from the data, which would have significantly reduced the sample size, was not justified in this instance.

In the future, with an ever-increasing amount of faunal isotope data and radiocarbon dates being published, it is hoped that species-specific geostatistical analyses can be further explored. Such investigations would undoubtedly be of great benefit to furthering our understanding the isotope ecology and niche overlap/partitioning of key herbivore species, and of the complex and competing influences of environment and ecology on faunal δ^15^N.

### 4.3 Isoscape mapping using faunal isotope data

This study represents the first (to our knowledge) study to create temporally-layered isoscapes using palaeo-data. While the application of isoscape approaches in modern terrestrial environmental and ecological research are now relatively widespread [27], the use of isoscape modelling in palaeo-focused research has so far been more limited [72–76]. In part, this can be attributed to the additional complexities that palaeo-isoscapes must contend with; while plant, soil or animal δ^15^N is a spatially and temporally continuous variable, our means of sampling such data is inescapably discretized (i.e. each sample represents a discrete temporal and spatial interval). The discretization is many orders of magnitude larger in fossil than in modern data ensembles, owing to the uncertainties in establishing calendar age estimates for fossil samples, the need to consider samples of different ages together as a single temporal unit, and the assumptions that must be made about the spatial resolution and provenance of the sample. As such, isoscapes constructed using palaeo-data will always have a certain level of unavoidable uncertainty inbuilt.

Likewise, probably owing to the complex nature of the terrestrial nitrogen cycle and relative data paucity compared to other environmental systems (e.g. oxygen and hydrogen in the hydrological cycle), the application of geostatistical approaches specifically toward terrestrial nitrogen isotope data have so far also been comparatively limited [5, 77–79]. Part of the difficulty in assessing regional/global scale gradients in δ^15^N is that the nitrogen isotope composition of soils and plants may be highly heterogeneous at very localised spatial and/or temporal scales [80]. In this regard, relying on bone collagen data may actually be advantageous; herbivores act as natural integrators, providing a measure of ecosystem nitrogen that is spatially averaged over the extent of the animal’s home range, and temporally averaged over a number of years (temporal resolution depends on bone collagen turnover rate, but is typically in the order of several years). Therefore, while the use of faunal δ^15^N to trace changes in underlying environmental δ^15^N introduces noise from dietary and behavioural differences, it also offers a unique means to assess ecosystem-scale variation in δ^15^N, particularly in past environments, where other sampling opportunities are lacking or inadequate.

A recent study by Barrientos et al. [73] illustrated the potential of using archaeological bone collagen δ^15^N data in palaeo-isoscape mapping. The resultant Inverse Distance Weighted (IDW) isoscapes demonstrated how geostatistical approaches, rooted in community and trophic ecology, could be applicable to addressing archaeological questions [73]. In this study we have progressed these ideas, demonstrating the possibility of applying a more complex geostatistical method, which also allows for errors and uncertainties to be quantified and covariate data to be incorporated. The approach followed here demonstrates one such method that allows consideration of variations in spatial gradients in δ^15^N through time. We chose to use a mixed model approach as mixed models are widely used in ecological studies, and it enabled spatially explicit variance surfaces to be calculated and spatial and environmental components to be fitted jointly [47, 81]. Alternative approaches could be used, for example a Bayesian hierarchical approach, such as integrated nested Laplace approximation, or machine learning approaches, such as random forest, cubist and stochastic gradient boosting [77, 82, 83]. Indeed, these approaches may allow us to further refine the δ^15^N palaeo-isoscapes and better account for uncertainty in the predictions. Of particular interest for future research will be to test the effect of incorporating additional random effects, such as species, time/age uncertainty, tissue type/skeletal element into the models.

Regardless of the modelling approach followed, the quality, quantity, and spatial/temporal distribution of the underlining data is key. While the prediction models presented here provide an estimate of site-averaged herbivore δ^15^N in the past, at locations where empirical data is absent, such isoscape approaches should not replace efforts to establish local and time-specific baseline data through empirical sampling. The two approaches need to work hand-in-hand, as continued efforts to generate empirical data will ultimately lead to improvements in the predictive power of isoscape models. Largescale data syntheses must contend with potential issues and uncertainties introduced by inconsistent reporting of data and associated metadata, for example, missing or incomplete information regarding analytical and laboratory protocols, sampling methods, data standardisation procedures, and basic sample metadata. Through recent efforts to create robust and standardized reporting guidelines and open access data repositories many of these issues are beginning to be addressed [84–86]. This will ultimately lead to improved data quality which will benefit all types of data analysis, including isoscape approaches.

## 5. Conclusion

Here we have presented time-sliced maps of terrestrial δ^15^N gradients based on archaeological and palaeontological animal isotope data. In addition to compiling and critically evaluating previously published data, our analysis includes the publication of several hundred new faunal δ^15^N data and radiocarbon dates. The analysis serves two main purposes; to investigate changes in spatial gradients of δ^15^N in late Pleistocene Europe, with a view to investigating the Late Glacial Nitrogen Excursion, and to demonstrate more broadly the application of isoscape approaches to palaeo-data with implications for how baseline data is understood and used in archaeological and palaeoecological research.

Our results have shown clear changes in spatial gradients of δ^15^N through time, that are most likely related to changes in landscape moisture (particularly from increased input of meltwater from icesheets and thawing permafrost) that occurred after the Last Glacial Maximum. Our analysis found that the inclusion of climatic covariate data in the models did not significantly improve model performance, suggesting that the combination of the variables considered did not fully capture the drivers producing the observed spatial variation in the δ^15^N faunal data.

The results highlight the significant opportunities and challenges of applying isoscape approaches to faunal data. With the continued publication of faunal isotope data from archaeological and palaeontological assemblages, it is likely that in the coming years the accuracy and the temporal and spatial resolutions of such models can be much improved upon. While the nature of fossil sample material dictates that there will always be limitations in the accuracy of such models, the value of such predictive maps for investigating long term continental-scale changes in the terrestrial nitrogen cycle, and natural- and anthropogenically-driven impacts on said cycle, should not be understated. Such models can make an important contribution to understanding baseline δ^15^N values for terrestrial food web analysis and in the interpretation of data from higher trophic level animals in relation to mobility, migration, and dietary research. Moreover, improved understanding of baseline δ^15^N in late Pleistocene and early Holocene contexts provides a background reference against which subsequent human impact on the nitrogen cycle and overall landscape health, such as through farming practices and deforestation, can be assessed.

## Supporting information

S1 Dataset (XLSX)

S2 Dataset (R)

S1 File (DOCX)

S2 File Base maps for figures. (ZIP)
