# Supplementary material for: Nitrogen palaeo-isoscapes: Changing spatial gradients of faunal δ15N in late Pleistocene and early Holocene Europe
Source: PLoS One. 2023 Feb 6;18(2):e0268607. doi: 10.1371/journal.pone.0268607 (PMC9901814; doi:10.1371/journal.pone.0268607)
Supplement: S1 File — (DOCX) [file pone.0268607.s003.docx]

# S3 Supporting Information

# Nitrogen palaeo-isoscapes:

# Changing spatial gradients of faunal δ^15^N in late Pleistocene and early Holocene Europe

Hazel Reade^1*^, Jennifer A. Tripp^1,#a^, Delphine Frémondeau^1^, Kerry L. Sayle^2^, Thomas F.G. Higham^3, #b, #c^, Martin Street^4^, Rhiannon E. Stevens^1^

^1^UCL Institute of Archaeology, University College London, London, United Kingdom.

^2^Scottish Universities Environmental Research Centre, , Glasgow, United Kingdom.

**^3^Research Laboratory for Archaeology and the History of Art, University of Oxford**, Oxford, United Kingdom.

^4^Römisch-Germanisches Zentralmuseum, Forschungsinstitut für Archäologie Kompetenzbereich Pleistozäne und Frühholozäne Archäologie, Neuwied, Germany.

^#a^Current address: Department of Chemistry, University of San Francisco, San Francisco USA

^#b^Current address: Department of Evolutionary Anthropology, Faculty of Life Sciences, University of Vienna, Vienna, Austria

^#c^Current address: Human Evolution and Archaeological Sciences (HEAS), University of Vienna, A-1030, Vienna, Austria.

* Corresponding author

Email: [h.reade@ucl.ac.uk](mailto:h.reade@ucl.ac.uk) (HR)

# 1 Sample preparation and isotope and radiocarbon analytical methods

Of the 717 newly generated $\delta$^15^N data reported in this study, 95 came from pre-existing collagen samples and 622 came from newly collected material from which we extracted the collagen. The pre-existing collagen samples were originally produced as part of other research projects, for example those focusing on radiocarbon dating, and the leftover collagen was kindly donated to this study for the purpose of $\delta$^15^N analysis.

For the newly collected material, a small (<1g) sample of bone or tooth dentine was taken from each specimen using a dental drill with either a small cutting wheel or tungsten burr attachment. This sample collection was conducted over many years and collagen extraction and isotope analysis was performed at different laboratories. The S1 Dataset file details which of the following collagen extraction methods were followed for each sample, and the analysing laboratory. Collagen extraction methodology is also given in the S1 Dataset file for data previously published in Stevens [1]. These samples were analysed at RLAHA, University of Oxford following the analytical methodology given in Stevens [1].

Collagen extracted at University College London (UCL) followed a modified version of the Oxford Radiocarbon Accelerator Unit (ORAU) collagen extraction procedures (AF and AG methods [2]), which is based on a modified version of the Longin method [3]. For samples that had been, or were suspected to have been, conserved with PVA glue, a solvent extraction pre-treatment was used to remove the adhesive (denoted as AG* or AF* method). All samples were demineralised in 0.5 M hydrochloric acid (HCl) at 4°C and then thoroughly rinsed with ultrapure water. Unless otherwise indicated in the Supplementary Data File, all samples were then treated with 0.1 M sodium hydroxide (NaOH) for 30 minutes to remove humic acids, before being thoroughly rinsed. Samples were then gelatinised in pH 3 HCl solution at 75°C for 48h and filtered using a pre-cleaned Ezee-filter (AG method). For some samples, including all those radiocarbon dated, the filtrate was then passed through a pre-cleaned 15–30 kD ultrafilter, with the > 30 kD fraction collected and freeze-dried (AF method).

Collagen extracted at RLAHA, University of Oxford also followed a modified version of the Longin method [3], which is detailed in Stevens and Hedges [4]. For samples that had been, or were suspected to have been, conserved with PVA glue, a solvent extraction pre-treatment was used to remove the adhesive (RLAHA Method 1 and 3 used this step). Solvent extraction involved heating the sample at 40°C for an hour in distilled water, then repeating the heating process using acetone, distilled water, methanol, and distilled water, respectively. Samples were then demineralised in 0.5 M aq. HCl at 4°C until the mineral fraction had dissolved and then rinsed three times with distilled water (RLAHA Methods 1-4 followed this step). Where sample amount was sufficient and where deemed necessary 0.1 M NaOH was added for 30 minutes to remove [humic acids](https://www.sciencedirect.com/topics/agricultural-and-biological-sciences/humic-acid) (RLAHA Method 1 and 2 used this step). Samples were then rinsed with distilled water, gelatinised in a pH 3 solution for 48 hours at 75 °C (RLAHA Methods 1-4 followed this step). The filtered supernate containing the soluble collagen was then collected, frozen, and lyophilized.

Collagen extracted at the University of Cambridge again followed a broadly similar method, which is detailed in Stevens et al [5]. Samples were demineralised in 0.5 M aq. HCl at 4 °C until they had fully demineralised. Samples were then rinsed in distilled water and gelatinised by heating in pH 3.0 aqueous solution at 75 °C for 48 h. The liquid fraction containing the dissolved collagen was filtered off, frozen overnight at −20°C, then stored at −80°C for 4 h and finally lyophilised.

Isotopic analysis at the Scottish Universities Environment Research Centre (SUERC) was undertaken using a Delta V Advantage continuous-flow isotope ratio mass spectrometer coupled via a ConfloIV to an IsoLink Elemental Analyser (Thermo Scientific, Bremen). Between 1.2 and 1.5mg collagen was loaded into a tin capsule for continuous flow combustion and isotopic analysis. For every ten archaeological samples, three in-house standards (SAG: δ^15^N_AIR_ = 4.3 ± 0.2‰, MAG: δ^15^N_AIR_ = 47.3 ± 0.1‰, and MSAG: δ^15^N_AIR_ = 3.1 ± 0.2‰), which are calibrated to the International Atomic Energy Agency (IAEA) reference materials USGS40 (δ^15^N_AIR_ = –4.5 ± 0.1‰), USGS41 (δ^15^N_AIR_ = 47.6 ± 0.2‰), and IAEA-N-1 (δ^15^N_AIR_ = 0.43 ± 0.1‰), were used to normalize the δ^15^N values [6]. Results are reported as per mil (‰) relative to the internationally accepted standard AIR. Normalization was checked using the well characterised Elemental Microanalysis IRMS fish gelatin standard B2215 (δ^15^N_AIR_ = 4.3 ± 0.1‰) and precision was determined to ± 0.2‰ for δ^15^N based on repeated measurements of an in-house bone collagen standard (DHB2019: δ^15^N_AIR_ = 3.7 ± 0.2‰) [6].

Isotopic analysis at the Godwin Laboratory, University of Cambridge was undertaken using an automated elemental analyser (Costech Analytical, Valencia, CA, USA) coupled in continuous-ﬂow mode to a Thermo Finnigan MAT253 isotope ratio mass spectrometer (Thermo Fisher Scientiﬁc, Bremen, Germany). Between 0.6 and 1 mg of collagen was loaded into a tin capsule for isotopic analysis. International (IAEA: caffeine and glutamic acid-USGS-40) standards with known isotopic values and in-house laboratory standards (nylon, alanine and bovine liver standards) calibrated to the IAEA standards were interspersed throughout each analytical run and were used to normalize the collagen δ^15^N values. Results are reported using the delta scale in per mil (‰) relative to the internationally accepted standard AIR. Samples were analysed in duplicate with δ^15^N analytical errors of ±0.2‰ based on repeated measurements of calibration standards.

In some instances, samples with previously published δ^15^N values have subsequently been re-analysed. For example, some samples with previously published δ^15^N values from the RLAHA laboratory [4,7], have subsequently been reanalysed at the SUERC laboratory as part of ongoing research focusing on the simultaneous analyses of δ^15^N, δ^13^C, and δ^34^S. Duplicate data is noted in the S1 Dataset.

Radiocarbon dating was performed at the Oxford Radiocarbon Accelerator Unit (ORAU) using their standard procedures [2]. Approximately 5 mg of dry collagen per sample was weighed into pre-baked tin capsules and combusted using an elemental analyser coupled to an isotope ratio mass spectrometer, employing a splitter to allow for collection of the CO_2_ [2,8]. Samples were graphitised by reduction of collected CO_2_ over an iron catalyst in an excess H_2_ atmosphere at 560 °C [9,10]. The ^14^C dates were measured on the Oxford AMS system using a caesium ion source for ionisation of the solid graphite sample [11]. To denote samples where collagen extraction took place at a laboratory other than ORAU, all measured dates were given “OxA-V-wwww-pp” numbers, where “wwww” indicates the wheel number, and “pp” is the position of the sample on the wheel [2]. For samples where collagen was extracted at UCL, following the method outlined by Wood et al [12], background corrections were applied to our dates to account for inter-laboratory differences in background carbon. A full description of our correction methodology is detailed in Reade et al [13] . Corrected dates are denoted by the “C” at the end of the date code assigned by ORAU. All dates are given as uncalibrated radiocarbon dates (^14^C BP) and calibrated dates BP (cal. BP) in the Supplementary Data file. Date calibration was performed using OxCal 4.4 [14] and the IntCal20 dataset [15].

# 2 Inter-species comparisons

While herbivore δ^15^N tracks that of the environmental baseline δ^15^N, differences in dietary behaviours between different species (and between different populations or individuals of the same species) introduce variation into the δ^15^N signal. Herbivore feeding habits typically fall on a spectrum between graze-dominated and browse-dominated diets; the position of a species on this continuum is partly determined by dietary physiology and partly determined by environmental conditions and inter-species competition in a given location/time context. Broadly, higher δ^15^N is associated with grazers and lower δ^15^N with browsers within a given ecosystem, although this pattern is not consistent through space or time [16–18]. Further variation may occur in herbivore δ^15^N related to the consumption of different plant parts and different plant species within the graze (typically grasses, sedges, forbs) or browse (typically shrubs and tree foliage) groupings [19]. Additionally, the proportion of different plant species consumed may not be represented in the bone collagen δ^15^N in directly equivalent proportions. As bone collagen δ^15^N predominately represents the δ^15^N of dietary proteins, for a species consuming a mix of protein-rich and protein-poor plant types, it is the protein-rich species that will be greater represented in the bone collagen δ^15^N signature [17].

The assembled data were evaluated for potential species-based effects related to diet, habitat preference, and ecology on δ^15^N. Each taxon was assigned to a dietary category (either grazer, browser, or mixed-feeder) based on prevailing understanding of dietary behaviour (see Schwartz-Narbonne et al [17] and references therein for detailed discussion). In summary, *Equus*, *Bos*/*Bison, Mammuthus* and *Coelodonta* were categorised as grazers, *Rangifer, Cervus elaphus*, *Megaloceros, Saiga, Ovibos* and *Rupicapra* were categorised as mixed feeders, and *Alces,* *Capra* and *Capreolus* were categorised as browsers. In making these categorisations the dietary behaviour that is considered most dominant, or most commonly evident in extant species, was selected. However, most species display considerable ecological flexibility, and dietary behaviours are strongly influenced by environmental conditions and inter-species competition [17,18].

Data was divided by time bin and δ^15^N was compared between species and dietary category (Fig S2.1). Kruskal-Wallis tests indicate significant differences in δ^15^N between species and in δ^15^N between dietary categories (Table S2.1). This is not unexpected as the comparison makes no consideration of the spatial distribution of different species and thus data spanning a range of climatic and environmental zones are also being compared.

**Table S2.1 Kruskall-Wallis test statistics comparing δ^15^N between species, and δ^15^N between dietary categories, for each time bin.**

| Time bin | Species Comparison | | Dietary Comparison | |
| --- | --- | --- | --- | --- |
|  | **Test statistic** | ***p*** | **Test statistic** | ***p*** |
| EH | 30.9 | <0.000 | 11.8 | 0.003 |
| YD | 11.3 | 0.003 | 12.5 | <0.000 |
| LGI | 88.0 | <0.000 | 22.2 | <0.000 |
| LGT | 274.0 | <0.000 | 77.3 | <0.000 |
| LGM | 57.3 | <0.000 | 5.91 | 0.052 |
| LOIS3 | 25.6 | <0.000 | 17.0 | <0.000 |
| EOIS3 | 49.9 | <0.000 | 20.5 | <0.000 |


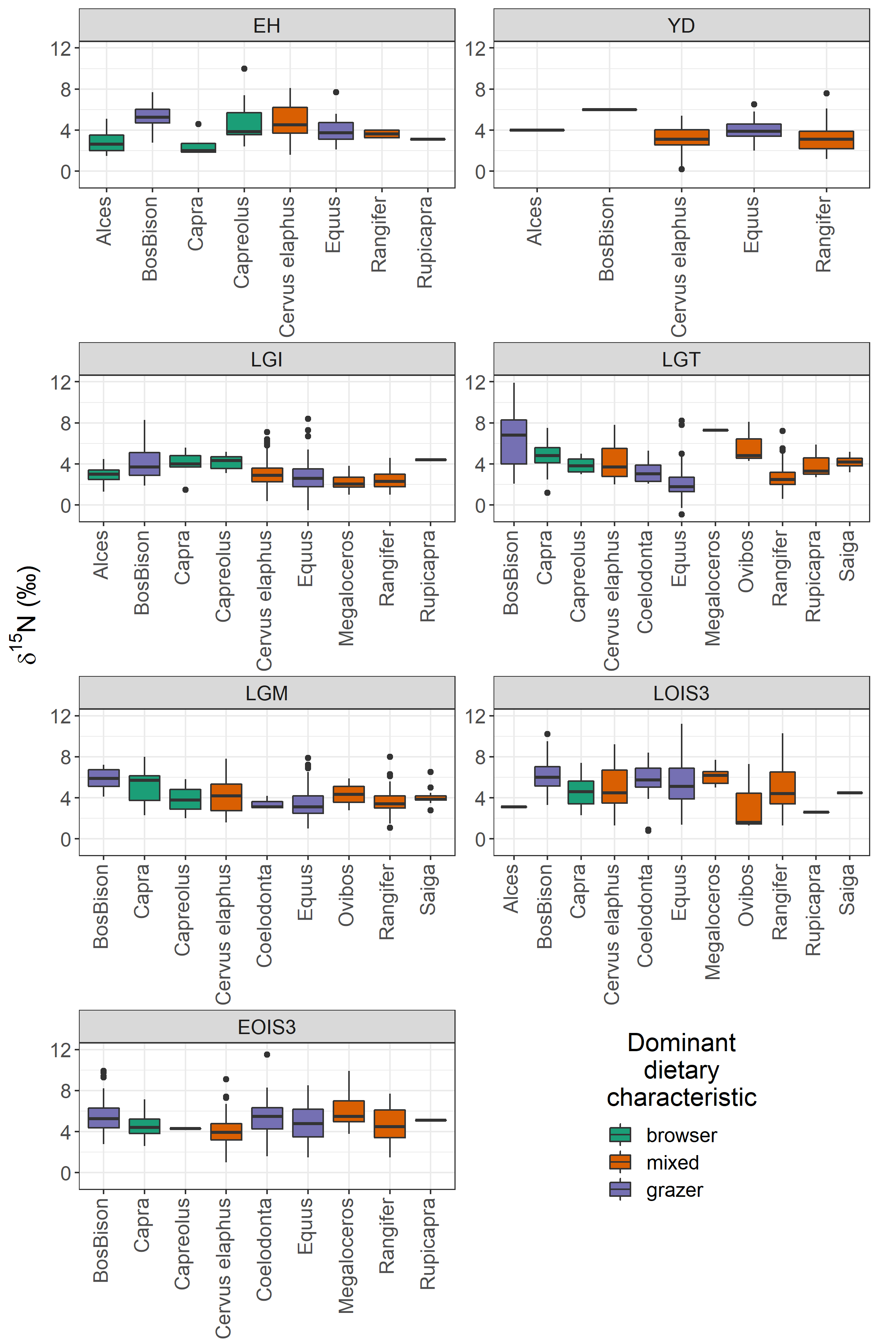


**Figure S2.1 Boxplots showing the range of δ^15^N values for each species, divided by time bin, and coloured by dominant dietary characteristic**

Differences between species can be more robustly examined by considering δ^15^N variability within spatiotemporal clusters. Samples within the same time bin which originated from locations within 100km of one another were grouped together in clusters. Inter-species differences were then investigated for each spatiotemporal cluster where at least 2 different species were present, each with at least 3 data points. Within-cluster δ^15^N was compared between species using Mann Whitney U tests where the number of species was 2 and using Kruskal-Wallis tests where the number of species were greater than 2. Of the 65 spatiotemporal clusters evaluated for species-based differences, 31 showed significant differences in δ^15^N between species and 34 did not (Table S2.2), and no consistent pattern in space or time is apparent (Fig S2.2). The is no relationship between the number of species per cluster and statistical difference between species δ^15^N, either when the data is considered whole (spearman rank correlation r=-0.167, p>0.05), or when divided by time bin (Table S2.3). There is a relationship between the number of samples per cluster and statistical difference between species δ^15^N (spearman rank correlation r=-0.396, p<0.05), but when the data is considered by time bin it is clear that this is being driven by the EOIS3 samples (Table S2.3), which we suggest highlights a potential data aggregation problem with this time bin.

**Table S2.2 Summary and test statistics for comparisons of δ^15^N between species by spatiotemporal clusters. Tests for difference were and Mann-Whitney (MW) where number of species = 2 and Kruskall-Wallis (KW) where n ≥ 3. Significance was taken to be *p* < 0.05 and significant differences are indicated with ***

| **Early Holocene** | | | | | | | | | | |
| --- | --- | --- | --- | --- | --- | --- | --- | --- | --- | --- |
| **Cluster** | **Faunal Category** | **n** | **δ^15^N (‰)** | | | | | **Test Statistic** | **P value** | **Test** |
|  |  |  | **Mean** | **Median** | **s.d.** | **Min** | **Max** |  |  |  |
| 1 | BosBison | 3 | 6 | 6 | 0.1 | 6 | 6.1 | 12.0 | 0.050* | MW |
|  | Capreolus | 4 | 3.4 | 3.5 | 0.4 | 2.9 | 3.8 |  |  |  |
| 2 | BosBison | 9 | 5.2 | 5.2 | 0.6 | 4.7 | 6.6 | 18.0 | 0.456 | MW |
|  | Equus | 3 | 4.2 | 2.7 | 3.1 | 2.1 | 7.7 |  |  |  |
| 5 | Capra | 3 | 1.9 | 1.9 | 0.2 | 1.8 | 2.1 | 0.0 | 0.050* | MW |
|  | Cervus elaphus | 4 | 3.8 | 4 | 0.4 | 3.2 | 4.1 |  |  |  |
| 7 | BosBison | 5 | 5.4 | 5.2 | 0.5 | 4.9 | 6 | 2.5 | 0.29 | KW |
|  | Capreolus | 7 | 5.8 | 5.5 | 2.1 | 3.7 | 10 |  |  |  |
|  | Cervus elaphus | 5 | 6.3 | 6.8 | 1.5 | 3.7 | 7.4 |  |  |  |
| 18 | BosBison | 3 | 5.5 | 5.3 | 0.4 | 5.2 | 5.9 | 24.0 | 0.012* | MW |
|  | Cervus elaphus | 8 | 4.2 | 4.2 | 0.6 | 3.4 | 5 |  |  |  |
| Younger Dryas | | | | | | | | | | |
| **Cluster** | **Faunal Category** | **n** | **δ^15^N (‰)** | | | | | **Test Statistic** | **P value** | **Test** |
|  |  |  | **Mean** | **Median** | **s.d.** | **Min** | **Max** |  |  |  |
| 1 | Equus | 12 | 4.4 | 4.4 | 0.7 | 3.6 | 5.8 | 128.0 | 0.583 | MW |
|  | Rangifer | 19 | 4.3 | 4.1 | 1.3 | 1.8 | 7.6 |  |  |  |
| 2 | Equus | 4 | 3.4 | 3.5 | 0.5 | 2.8 | 3.8 | 10.5 | 0.154 | MW |
|  | Rangifer | 3 | 2.7 | 2.8 | 0.5 | 2.2 | 3.1 |  |  |  |
| 3 | Equus | 3 | 3.7 | 4.3 | 1.1 | 2.5 | 4.4 | 38.0 | 0.073 | MW |
|  | Rangifer | 15 | 2.5 | 2.4 | 0.9 | 1.3 | 5 |  |  |  |
| 5 | Equus | 4 | 3.3 | 3.2 | 0.5 | 2.9 | 3.9 | 38.5 | 0.011* | MW |
|  | Rangifer | 10 | 2 | 1.9 | 0.6 | 1.2 | 3 |  |  |  |
| Late Glacial Interstadial | | | | | | | | | | |
| **Cluster** | **Faunal Category** | **n** | **δ^15^N (‰)** | | | | | **Test Statistic** | **P value** | **Test** |
|  |  |  | **Mean** | **Median** | **s.d.** | **Min** | **Max** |  |  |  |
| 1 | BosBison | 3 | 5.5 | 5.7 | 0.6 | 4.9 | 6 | 20.2 | <0.000* | KW |
|  | Capreolus | 7 | 4.6 | 4.8 | 0.6 | 3.2 | 5.2 |  |  |  |
|  | Cervus elaphus | 11 | 4.5 | 4.5 | 0.9 | 2.9 | 5.5 |  |  |  |
|  | Equus | 7 | 3.8 | 3.6 | 0.5 | 3 | 4.4 |  |  |  |
|  | Rangifer | 19 | 3.5 | 3.4 | 0.7 | 2.3 | 4.6 |  |  |  |
| 2 | Alces | 14 | 2.8 | 2.7 | 0.6 | 1.3 | 3.6 | 2.6 | 0.45 | KW |
|  | BosBison | 13 | 3.1 | 2.9 | 0.6 | 2.1 | 4.2 |  |  |  |
|  | Cervus elaphus | 29 | 3.1 | 3.3 | 0.7 | 1.7 | 4.4 |  |  |  |
|  | Equus | 3 | 3.2 | 2.5 | 1.6 | 2.1 | 5.1 |  |  |  |
| 4 | BosBison | 15 | 3.5 | 3.6 | 0.6 | 2.6 | 4.6 | 41.0 | 0.033* | MW |
|  | Equus | 3 | 1.8 | 1.8 | 1.4 | 0.4 | 3.1 |  |  |  |
| 6 | Cervus elaphus | 6 | 3.4 | 3.5 | 0.4 | 2.8 | 4 | 0.9 | 0.623 | KW |
|  | Equus | 11 | 3.3 | 3.2 | 1 | 2 | 5.4 |  |  |  |
|  | Rangifer | 8 | 3.1 | 3.1 | 0.5 | 2.3 | 3.9 |  |  |  |
| 7 | Capra | 5 | 4.5 | 4.2 | 0.8 | 3.7 | 5.6 | 0.5 | 0.051 | MW |
|  | Cervus elaphus | 3 | 5.8 | 5.8 | 0.2 | 5.6 | 6 |  |  |  |
| 10 | Alces | 5 | 3.8 | 4 | 0.7 | 2.8 | 4.5 | 5.4 | 0.144 | KW |
|  | BosBison | 11 | 4.7 | 4.9 | 1 | 2.4 | 5.8 |  |  |  |
|  | Cervus elaphus | 7 | 4 | 4.2 | 0.6 | 3.2 | 4.7 |  |  |  |
|  | Equus | 10 | 4.1 | 4.2 | 1.1 | 2.5 | 5.3 |  |  |  |
| 12 | Equus | 4 | 2.2 | 2.4 | 0.6 | 1.5 | 2.7 | 53.5 | 0.221 | MW |
|  | Rangifer | 19 | 1.8 | 1.8 | 0.5 | 1 | 3.2 |  |  |  |
| 14 | Equus | 19 | 2.9 | 3 | 0.6 | 1.8 | 4 | 179.0 | 0.096 | MW |
|  | Rangifer | 14 | 2.6 | 2.6 | 0.4 | 1.6 | 3.1 |  |  |  |
| 15 | BosBison | 4 | 7.1 | 7.1 | 1 | 6 | 8 | 8.0 | 1 | MW |
|  | Equus | 4 | 6.8 | 7 | 1.5 | 4.9 | 8.4 |  |  |  |
| 19 | Cervus elaphus | 26 | 2.4 | 2.3 | 0.5 | 1.7 | 4.2 | 176.5 | <0.000* | MW |
|  | Equus | 7 | 1.4 | 1.4 | 0.4 | 0.8 | 1.9 |  |  |  |
| 20 | Capreolus | 8 | 4 | 4 | 0.5 | 3.2 | 4.6 | 36.5 | 0.019* | MW |
|  | Cervus elaphus | 5 | 3 | 3.1 | 0.5 | 2.2 | 3.6 |  |  |  |
| 22 | BosBison | 3 | 3 | 2.8 | 0.3 | 2.8 | 3.4 | 7.6 | 0.022* | KW |
|  | Cervus elaphus | 3 | 1.8 | 1.8 | 0.5 | 1.3 | 2.2 |  |  |  |
|  | Rangifer | 13 | 1.6 | 1.5 | 0.4 | 1.3 | 2.5 |  |  |  |
| 23 | Cervus elaphus | 17 | 2 | 2 | 0.7 | 0.4 | 3.1 | 50.0 | 0.567 | MW |
|  | Rangifer | 7 | 2.2 | 2.3 | 0.5 | 1.3 | 2.8 |  |  |  |
| 29 | Equus | 11 | 2.5 | 2.5 | 0.8 | 1 | 3.9 | 22.0 | 0.436 | MW |
|  | Rangifer | 3 | 2.2 | 2.2 | 0.4 | 1.8 | 2.6 |  |  |  |
| Last Glacial Termination | | | | | | | | | | |
| **Cluster** | **Faunal Category** | **n** | **δ^15^N (‰)** | | | | | **Test Statistic** | **P value** | **Test** |
|  |  |  | **Mean** | **Median** | **s.d.** | **Min** | **Max** |  |  |  |
| 1 | BosBison | 3 | 2.6 | 2.8 | 0.4 | 2.2 | 2.9 | 5.8 | 0.121 | KW |
|  | Coelodonta | 3 | 2.7 | 2.9 | 0.6 | 2.1 | 3.2 |  |  |  |
|  | Equus | 75 | 2.1 | 1.9 | 0.9 | -0.9 | 3.7 |  |  |  |
|  | Rangifer | 32 | 1.9 | 1.9 | 0.6 | 0.6 | 3 |  |  |  |
| 2 | BosBison | 4 | 3.4 | 2.8 | 1.7 | 2.1 | 5.7 | 7.1 | 0.029* | KW |
|  | Equus | 13 | 1.6 | 1.6 | 0.6 | 0.8 | 2.4 |  |  |  |
|  | Rangifer | 14 | 2.2 | 2.1 | 1 | 1.1 | 4.3 |  |  |  |
| 4 | Equus | 5 | 1.5 | 1.6 | 0.6 | 0.6 | 2.3 | 18.0 | 0.014* | MW |
|  | Rangifer | 25 | 2.3 | 2.4 | 0.5 | 1.2 | 3.4 |  |  |  |
| 6 | Capra | 28 | 5.3 | 5 | 1.1 | 3.9 | 7.5 | 13.6 | 0.001* | KW |
|  | Cervus elaphus | 42 | 5.5 | 5.5 | 0.7 | 3.9 | 7.2 |  |  |  |
|  | Equus | 4 | 3.4 | 3.4 | 0.3 | 3.1 | 3.7 |  |  |  |
| 7 | Equus | 4 | 3.7 | 3.7 | 0.9 | 2.7 | 4.6 | 5.8 | 0.056 | KW |
|  | Rangifer | 9 | 3.4 | 3.3 | 0.6 | 2.5 | 4.2 |  |  |  |
|  | Saiga | 3 | 4.7 | 4.5 | 0.5 | 4.3 | 5.2 |  |  |  |
| 8 | Equus | 8 | 1.7 | 1.8 | 0.3 | 1.2 | 2 | 0.0 | 0.008* | MW |
|  | Rangifer | 4 | 2.6 | 2.5 | 0.4 | 2.2 | 3.1 |  |  |  |
| 9 | Equus | 13 | 1.7 | 1.5 | 0.5 | 1.1 | 2.9 | 3.0 | 0.010* | MW |
|  | Rangifer | 4 | 2.9 | 2.8 | 0.3 | 2.6 | 3.2 |  |  |  |
| 10 | BosBison | 5 | 6 | 5.8 | 0.5 | 5.4 | 6.8 | 19.1 | 0.000* | KW |
|  | Equus | 5 | 4.2 | 4.2 | 0.5 | 3.4 | 4.8 |  |  |  |
|  | Rangifer | 10 | 3.5 | 3.6 | 0.6 | 2.4 | 4.6 |  |  |  |
|  | Saiga | 19 | 4.2 | 4.1 | 0.5 | 3.2 | 5.1 |  |  |  |
| 11 | BosBison | 24 | 8.2 | 8.1 | 1.4 | 6.1 | 11.9 | 311.0 | <0.000* | MW |
|  | Cervus elaphus | 15 | 6.5 | 6.5 | 0.8 | 5 | 7.8 |  |  |  |
| 13 | Cervus elaphus | 3 | 2.3 | 2.3 | 0.2 | 2.1 | 2.4 | 54.0 | 0.031* | MW |
|  | Equus | 20 | 1.2 | 1.1 | 0.8 | -0.3 | 3.2 |  |  |  |
| 18 | Equus | 17 | 2.8 | 2.1 | 1.7 | 1.3 | 8.2 | 6.6 | 0.037* | KW |
|  | Ovibos | 3 | 5.7 | 4.8 | 2.1 | 4.3 | 8.1 |  |  |  |
|  | Rangifer | 10 | 2.5 | 2.5 | 0.8 | 1.1 | 4.1 |  |  |  |
| Last Glacial Maximum | | | | | | | | | | |
| **Cluster** | **Faunal Category** | **n** | **δ^15^N (‰)** | | | | | **Test Statistic** | **P value** | **Test** |
|  |  |  | **Mean** | **Median** | **s.d.** | **Min** | **Max** |  |  |  |
| 2 | BosBison | 6 | 4.6 | 4.5 | 0.4 | 4.1 | 5.2 | 14.5 | 0.002* | MW |
|  | Equus | 20 | 3.2 | 3.2 | 1.1 | 1 | 6 |  |  |  |
|  | Rangifer | 48 | 3.4 | 3.3 | 0.8 | 1.5 | 5.2 |  |  |  |
|  | Saiga | 10 | 3.9 | 3.9 | 0.6 | 2.8 | 5 |  |  |  |
| 4 | Cervus elaphus | 46 | 3.2 | 2.8 | 1.3 | 1.6 | 7.8 | 15.5 | <0.000* | MW |
|  | Rangifer | 6 | 5.7 | 5.4 | 1.2 | 4.7 | 8 |  |  |  |
| 5 | Capra | 12 | 5.8 | 5.8 | 1.2 | 3.7 | 8 | 261.5 | 0.213 | MW |
|  | Cervus elaphus | 35 | 5.3 | 5.3 | 1.1 | 3.1 | 7.2 |  |  |  |
| 6 | Cervus elaphus | 7 | 4.8 | 4.6 | 0.5 | 4 | 5.7 | 35.0 | 0.005* | MW |
|  | Equus | 5 | 3 | 3.2 | 0.3 | 2.6 | 3.3 |  |  |  |
| 7 | BosBison | 3 | 6.9 | 6.8 | 0.2 | 6.8 | 7.1 | 0.3 | 0.843 | KW |
|  | Cervus elaphus | 6 | 6.4 | 6.7 | 1.1 | 5 | 7.5 |  |  |  |
|  | Equus | 7 | 7 | 6.9 | 0.5 | 6.5 | 7.9 |  |  |  |
| 8 | Equus | 4 | 2.7 | 2.7 | 0.9 | 1.8 | 3.7 | 3.5 | 0.14 | MW |
|  | Rangifer | 5 | 3.7 | 3.7 | 0.5 | 2.9 | 4.3 |  |  |  |
| 10 | BosBison | 8 | 6 | 6 | 0.6 | 5 | 6.9 | 17.5 | 0.305 | MW |
|  | Equus | 3 | 5.5 | 5.2 | 0.5 | 5.2 | 6.1 |  |  |  |
| 12 | Equus | 3 | 3.3 | 2.2 | 1.8 | 2.2 | 5.4 | 5.0 | 1 | MW |
|  | Rangifer | 3 | 2.5 | 2.6 | 1.4 | 1.1 | 3.9 |  |  |  |
| Late OIS 3 | | | | | | | | | | |
| **Cluster** | **Faunal Category** | **n** | **δ^15^N (‰)** | | | | | **Test Statistic** | **P value** | **Test** |
|  |  |  | **Mean** | **Median** | **s.d.** | **Min** | **Max** |  |  |  |
| 1 | BosBison | 4 | 5.4 | 5.0 | 1.5 | 4.3 | 7.5 | 6.4 | 0.094 | KW |
|  | Cervus elaphus | 10 | 5.4 | 4.7 | 1.8 | 3.8 | 8.5 |  |  |  |
|  | Equus | 34 | 6.5 | 6.5 | 2.3 | 3.0 | 11.2 |  |  |  |
|  | Rangifer | 28 | 7.1 | 7.3 | 1.7 | 4.1 | 10.3 |  |  |  |
| 2 | Cervus elaphus | 50 | 4.7 | 4.0 | 1.9 | 1.3 | 9.2 | 807.0 | 0.041* | MW |
|  | Equus | 25 | 3.8 | 3.6 | 1.8 | 1.4 | 8.0 |  |  |  |
| 3 | BosBison | 6 | 6.6 | 6.2 | 1.2 | 5.3 | 8.6 | 5.0 | 0.364 | MW |
|  | Coelodonta | 3 | 7.5 | 7.1 | 0.8 | 7.1 | 8.4 |  |  |  |
| 4 | BosBison | 5 | 4.6 | 4.2 | 1.5 | 3.3 | 6.8 | 10.7 | 0.005* | KW |
|  | Equus | 23 | 5.1 | 5.0 | 1.5 | 2.5 | 9.8 |  |  |  |
|  | Rangifer | 24 | 4.0 | 4.0 | 1.1 | 2.6 | 8.0 |  |  |  |
| 5 | Equus | 21 | 6.9 | 6.8 | 1.9 | 4.1 | 9.7 | 106.5 | 0.012* | MW |
|  | Rangifer | 6 | 4.4 | 4.6 | 1.0 | 2.6 | 5.8 |  |  |  |
| 6 | BosBison | 5 | 4.5 | 4.1 | 0.8 | 3.8 | 5.7 | 18.6 | <0.000* | KW |
|  | Coelodonta | 7 | 5.9 | 5.9 | 1.2 | 4.3 | 7.3 |  |  |  |
|  | Equus | 15 | 5.8 | 5.8 | 1.0 | 3.8 | 7.5 |  |  |  |
|  | Rangifer | 19 | 3.9 | 3.4 | 1.5 | 2.1 | 8.1 |  |  |  |
| 7 | BosBison | 8 | 5.5 | 5.6 | 0.6 | 4.6 | 6.1 | 19.0 | 0.67 | MW |
|  | Rangifer | 4 | 5.3 | 4.9 | 1.8 | 3.7 | 7.6 |  |  |  |
| 9 | Cervus elaphus | 5 | 4.8 | 4.6 | 1.4 | 3.2 | 7.1 | 4.1 | 0.126 | KW |
|  | Equus | 7 | 4.8 | 3.9 | 2.1 | 2.1 | 7.9 |  |  |  |
|  | Rangifer | 23 | 6.1 | 6.1 | 1.5 | 3.4 | 8.6 |  |  |  |
| 11 | BosBison | 7 | 6.8 | 6.1 | 1.7 | 5.0 | 9.3 | 0.6 | 0.759 | KW |
|  | Capra | 3 | 5.9 | 6.0 | 1.5 | 4.4 | 7.3 |  |  |  |
|  | Cervus elaphus | 4 | 6.3 | 6.0 | 1.0 | 5.5 | 7.7 |  |  |  |
| 12 | BosBison | 4 | 5.4 | 5.4 | 0.6 | 4.7 | 6.1 | 9.8 | 0.020* | KW |
|  | Coelodonta | 3 | 5.3 | 5.1 | 0.9 | 4.6 | 6.3 |  |  |  |
|  | Equus | 4 | 5.7 | 5.3 | 2.1 | 3.6 | 8.6 |  |  |  |
|  | Rangifer | 6 | 3.3 | 3.2 | 0.8 | 2.4 | 4.4 |  |  |  |
| 16 | BosBison | 8 | 7.5 | 8.2 | 2.3 | 3.5 | 10.2 | 14.6 | 0.002* | KW |
|  | Coelodonta | 3 | 5.4 | 5.4 | 0.1 | 5.3 | 5.5 |  |  |  |
|  | Equus | 3 | 4.3 | 3.8 | 1.9 | 2.6 | 6.4 |  |  |  |
|  | Rangifer | 8 | 2.6 | 2.8 | 0.8 | 1.3 | 3.6 |  |  |  |
| Early OIS 3 | | | | | | | | | | |
| **Cluster** | **Faunal Category** | **n** | **δ^15^N (‰)** | | | | | **Test Statistic** | **P value** | **Test** |
|  |  |  | **Mean** | **Median** | **s.d.** | **Min** | **Max** |  |  |  |
| 1 | Cervus elaphus | 3 | 3.6 | 3.8 | 0.4 | 3.1 | 3.9 | 2.8 | 0.252 | KW |
|  | Equus | 3 | 4.4 | 4.8 | 2.0 | 2.2 | 6.2 |  |  |  |
|  | Rangifer | 3 | 5.1 | 5.2 | 0.3 | 4.7 | 5.3 |  |  |  |
| 2 | Cervus elaphus | 45 | 3.5 | 3.2 | 1.2 | 1.9 | 9.1 | 348.0 | 0.245 | MW |
|  | Equus | 19 | 4.1 | 3.6 | 1.7 | 1.5 | 7.6 |  |  |  |
| 3 | BosBison | 4 | 5.3 | 5.5 | 0.8 | 4.1 | 6.0 | 10.2 | 0.017* | KW |
|  | Cervus elaphus | 19 | 5.1 | 5.1 | 1.0 | 3.7 | 7.3 |  |  |  |
|  | Equus | 21 | 4.5 | 4.3 | 1.6 | 1.6 | 6.7 |  |  |  |
|  | Rangifer | 11 | 6.3 | 6.5 | 1.2 | 3.4 | 7.7 |  |  |  |
| 4 | Cervus elaphus | 5 | 4.2 | 4.4 | 0.9 | 3.0 | 5.3 | 0.3 | 0.865 | KW |
|  | Equus | 7 | 4.6 | 4.7 | 1.8 | 2.1 | 6.5 |  |  |  |
|  | Rangifer | 5 | 4.2 | 4.6 | 0.7 | 3.3 | 4.8 |  |  |  |
| 5 | Coelodonta | 11 | 6.4 | 6.1 | 2.1 | 3.7 | 11.5 | 13.0 | 0.64 | MW |
|  | Equus | 3 | 6.8 | 6.4 | 1.4 | 5.6 | 8.4 |  |  |  |
| 6 | BosBison | 10 | 5.6 | 5.4 | 1.0 | 4.2 | 8.0 | 8.8 | 0.033* | KW |
|  | Cervus elaphus | 5 | 4.3 | 4.2 | 1.0 | 3.0 | 5.4 |  |  |  |
|  | Equus | 6 | 5.2 | 5.6 | 1.3 | 2.9 | 6.4 |  |  |  |
|  | Rangifer | 3 | 6.4 | 6.3 | 0.2 | 6.3 | 6.6 |  |  |  |
| 7 | BosBison | 5 | 7.6 | 8.1 | 1.3 | 6.3 | 9.3 | 8.6 | 0.013* | KW |
|  | Equus | 30 | 5.3 | 5.4 | 1.4 | 2.3 | 8.5 |  |  |  |
|  | Rangifer | 30 | 5.1 | 4.8 | 1.4 | 2.6 | 7.6 |  |  |  |
| 8 | BosBison | 3 | 3.2 | 3.3 | 0.4 | 2.8 | 3.6 | 2.9 | 0.575 | KW |
|  | Coelodonta | 5 | 3.6 | 4.2 | 1.1 | 2.2 | 4.8 |  |  |  |
|  | Equus | 18 | 4.0 | 3.7 | 1.8 | 1.7 | 7.3 |  |  |  |
|  | Megaloceros | 3 | 4.4 | 4.6 | 0.5 | 3.8 | 4.7 |  |  |  |
|  | Rangifer | 9 | 3.3 | 3.3 | 0.5 | 2.0 | 3.8 |  |  |  |
| 11 | BosBison | 21 | 5.4 | 4.9 | 1.8 | 3.4 | 9.9 | 2.2 | 0.336 | KW |
|  | Capra | 7 | 5.0 | 4.6 | 1.3 | 3.8 | 7.1 |  |  |  |
|  | Cervus elaphus | 6 | 4.3 | 4.1 | 1.5 | 2.7 | 6.6 |  |  |  |
| 12 | BosBison | 9 | 5.0 | 4.8 | 0.9 | 4.3 | 7.0 | 10.3 | 0.016* | KW |
|  | Coelodonta | 8 | 6.2 | 6.0 | 0.9 | 5.3 | 7.5 |  |  |  |
|  | Equus | 14 | 5.4 | 5.1 | 1.3 | 3.1 | 7.3 |  |  |  |
|  | Rangifer | 6 | 3.8 | 3.5 | 2.1 | 1.5 | 7.7 |  |  |  |
| 13 | Capra | 5 | 5.0 | 4.4 | 1.2 | 3.6 | 6.6 | 4.5 | 0.451 | MW |
|  | Cervus elaphus | 3 | 5.7 | 6.0 | 0.8 | 4.8 | 6.2 |  |  |  |
| 17 | Equus | 3 | 4.2 | 4.8 | 1.6 | 2.3 | 5.4 | 7.0 | 1 | MW |
|  | Rangifer | 5 | 4.8 | 4.4 | 1.3 | 3.2 | 6.8 |  |  |  |


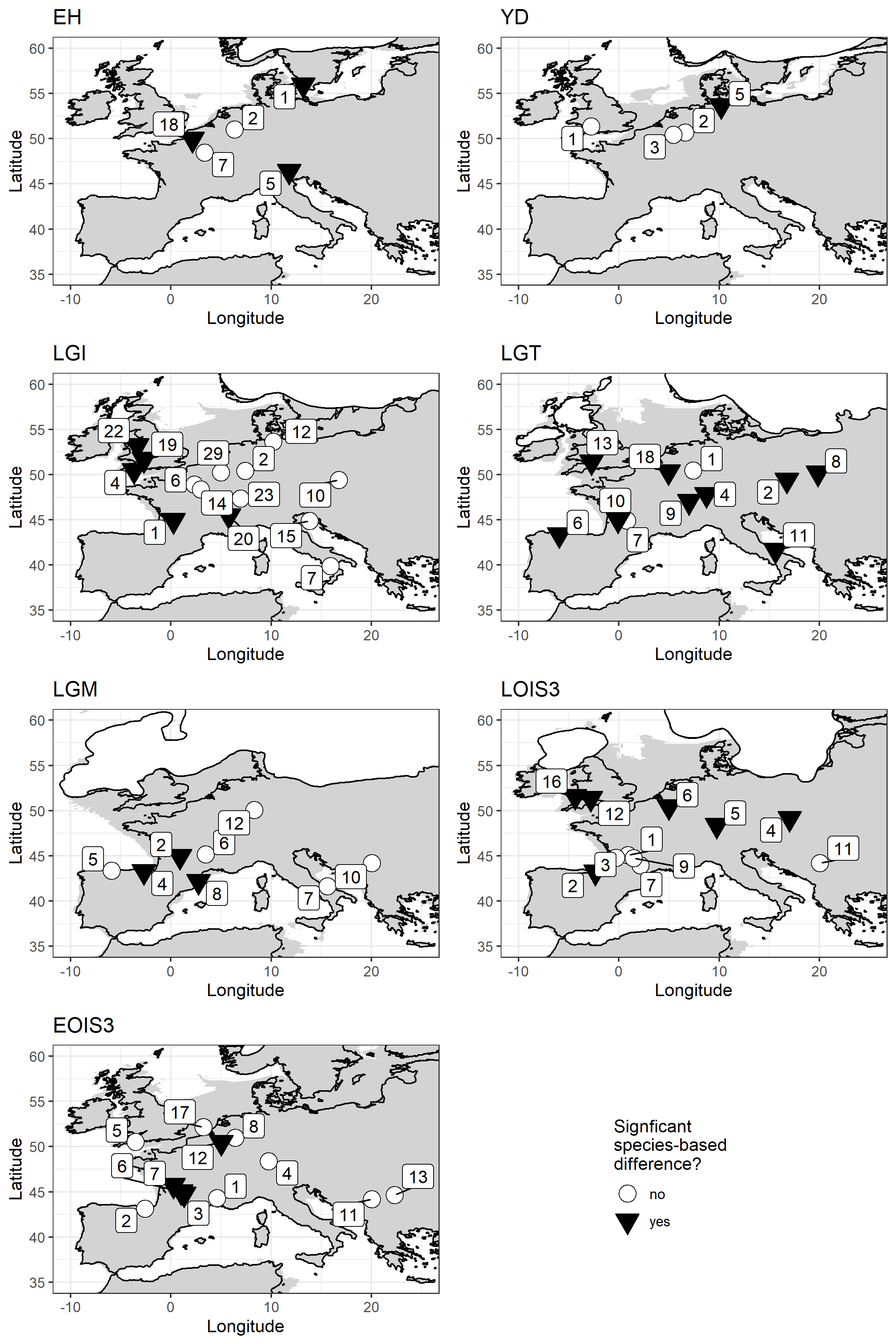


**Fig S2.2 Distribution of clustered samples. Each cluster contains samples from locations within a 100km search radii. Only clusters with at least two different species, with each species containing a minimum of three δ^15^N data points, are included. Symbols indicate clusters where δ^15^N differs significantly (p< 0.05) between species (black triangles) and clusters where it does not (white circles). Cluster numbers correspond to those given in Table S2.2. Palaeocoastline data from** [20]**, ice sheet extent from** [21] **and modern coastline from** [22].

**Table S2.3 Summary of significance testing for correlation between sample numbers/species numbers per cluster and statistical significance of between species d15N differences based on Spearman’s rank correlation. *indicates a significant relationship.**

| Time bin | Significance of correlation between inter-species d15N difference and number of samples per cluster | Significance of correlation between inter-species d15N difference and number of species per cluster |
| --- | --- | --- |
| EH | 0.306 | 0.549 |
| YD | 0.750 | - |
| LGI | 0.667 | 0.820 |
| LGT | 0.648 | 0.445 |
| LGM | 0.083 | 0.855 |
| LOIS3 | 0.146 | 0.143 |
| EOIS3 | 0.015* | 0.162 |

The lack of consistent species-based differences can be most clearly demonstrated by considering horse and reindeer data; these two species comprise 49% of the total data and both have wide geographical distributions across the study area (the exception being reindeer during the early Holocene). Horse and reindeer are known to have different dietary behaviours and thus would be expected to possess different δ^15^N signatures when occupying the same environment; horse are typically considered to be predominantly grazers, while reindeer are considered to be mixed feeders consuming a range of both browse and graze, as well as lichen [23–26]. These different plant types have different albeit overlapping and variable δ^15^N compositions [17,27].

In our data, horse and reindeer occur together in 35 spatiotemporal clusters. The difference in mean δ^15^N between these species within different spatiotemporal clusters ranges from ‑2.4‰ to +1.7‰ (mean = -0.3 ± 1.0 ‰), and a significant difference is identified in only 10 of the 35 clusters (Fig S4.3). The lack of systematic difference between these two species highlights the challenges faced when attempts are made to quantify and account for species-based differences. Indeed, while horse and reindeer and most commonly referred to as grazers and mixed-feeders respectively, a diversity of dietary behaviours are observed in extant populations of these species and evidenced in fossil assemblages (see discussion in Schwartz-Narbonne [17]. The lack of consistent differences between these two species most likely indicates dietary flexibility, with both species varying their diets relative to the availability of vegetation in their local environment.

Thus, in summary, while differences certainly occur in δ^15^N between species and dietary behaviours, we judge that there is too much variation in inter-species differences to enable adequate data normalisation/correction. By avoiding the use of such a correction our data retains a certain degree of noise associated with species/dietary differences, which may increase uncertainties associated with the geostatistical interpolations presented in the main manuscript. However, we believe the approach provides a more faithful representation of average baseline δ^15^N values and variability at the landscape scale. Species-specific geostatistical interpolations are considered in the discussion section of the main manuscript.


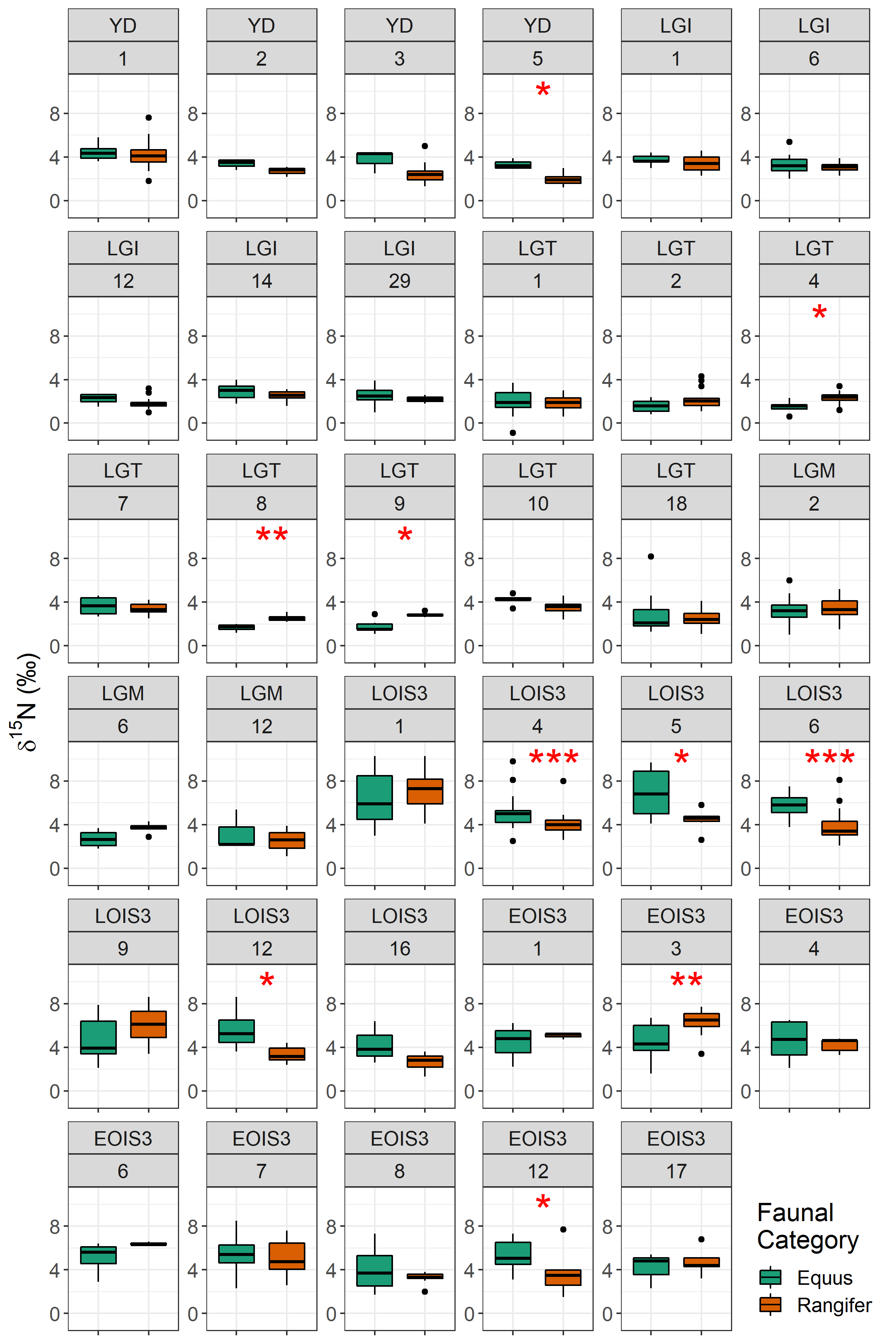


**Fig S2.3 Comparison of horse and reindeer δ^15^N values by spatiotemporal cluster. Significance indicated by: ****p* < .001, ** *p* < .01, **p* < .05.**

# 3 Summary of data, spatial autocorrelation and outlier analysis

**Table S3.1 Summary of faunal δ^15^N by time bin**

| **Age Bin (abbreviation)** | **n** | **Mean** | **Standard deviation** | **Median** | **Minimum** | **Maximum** |
| --- | --- | --- | --- | --- | --- | --- |
| Early Holocene (EH) | 176 | 4.7 | 1.6 | 4.6 | 1.5 | 10 |
| Younger Dryas (YD) | 133 | 3.4 | 1.3 | 3.4 | 0.2 | 7.6 |
| Late Glacial Interstadial (LGI) | 485 | 3.1 | 1.4 | 2.9 | -0.5 | 8.4 |
| Last Glacial Termination (LGT) | 602 | 3.3 | 1.9 | 2.9 | -0.9 | 11.9 |
| Last Glacial Maximum (LGM) | 339 | 4 | 1.5 | 3.8 | 1 | 8 |
| Late OIS 3 (LOIS3) | 465 | 5.3 | 2 | 5 | 0.8 | 11.2 |
| Early OIS 3 (EOIS3) | 518 | 4.7 | 1.6 | 4.5 | 1 | 11.5 |

**
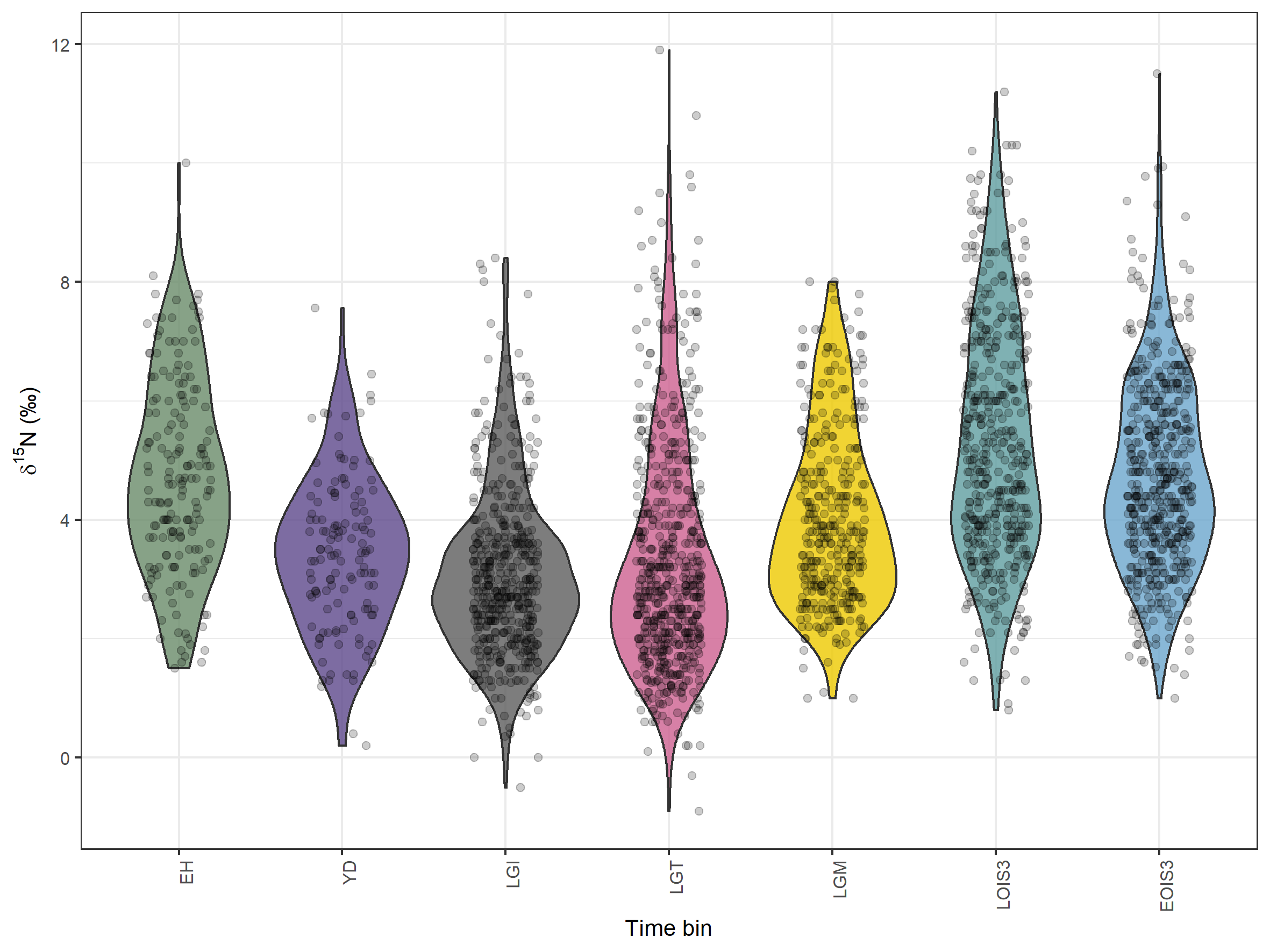
**

**Figure S3.1 Violin plot with overlying data points showing distribution of faunal δ^15^N data, plotted by time bin.**

**Table S3.2 Global Moran’s I test statistics and number of spatial outliers identified for each time bin using Anselin’s Local Moran’s I.**

| **Time bin** | **n** | **Number of spatial outliers** | **Global Moran’s I** | | | |
| --- | --- | --- | --- | --- | --- | --- |
|  |  |  | **Index** | **Expected Index** | **Z score** | **P value** |
| EH | 176 | 2 | 0.308 | -0.006 | 9.5 | <0.000 |
| YD | 133 | 15 | 0.498 | -0.009 | 12.6 | <0.000 |
| LGI | 485 | 18 | 0.515 | -0.002 | 35.9 | <0.000 |
| LGT | 602 | 33 | 0.665 | -0.002 | 55.2 | <0.000 |
| LGM | 339 | 25 | 0.521 | -0.003 | 28.7 | <0.000 |
| LOIS3 | 465 | 33 | 0.305 | -0.002 | 20.2 | <0.000 |
| EOIS3 | 518 | 60 | 0.305 | -0.002 | 21.1 | <0.000 |

To explore whether the number of outliers relates to our data aggregation procedures we first calculate the ratio of the number of outliers to the total number of samples for each time bin. We then compare that ratio to the length of time covered by each time bin and with the number of climatic oscillations per time bin (Table S3.3), defined as the number of Greenland Stadial and Greenland Interstadials determined by Rasmussen et al., 2014. No correlation exists between the outlier ratio and either parameter (t=0.73 and t=1.21, *p*>0.05, respectively). We therefore suggest that no single reason for number of outliers by time bin can be invoked, and we cannot discern between causes related to our data aggregation procedures and true localised environmental variability in δ^15^N and/or differences in animal ecology. For example, the relatively high ratio of outliers for the YD and EOIS3 time bins likely have different causes. For the YD, given that the time bin spans only 1,200 years, there is a much higher probability of inclusion of samples with an incorrect age assignment than for the EOIS3 time bin which spans 10,150 years. Conversely, for EOIS3, which spans 12 climatic ‘events’, there is a much higher probability that samples represent different climatic/environment states than those in the YD time bin, which should represent only 1 if all samples are correctly age-assigned.

**Table S3.3 Number of outliers, time span, and number of Greenland Stadial/Interstadial events per time bin.**

| **Time bin** | **n** | **Number of spatial outliers** | **Outlier ratio** | **Time bin span (years)** | **Number of GI and GS 'events'** |
| --- | --- | --- | --- | --- | --- |
| EH | 176 | 2 | 0.0114 | 3,460 | 1 |
| YD | 133 | 15 | 0.1128 | 1,200 | 1 |
| LGI | 485 | 18 | 0.0371 | 1,800 | 1 |
| LGT | 602 | 33 | 0.0548 | 4,850 | 1 |
| LGM | 339 | 25 | 0.0737 | 8,000 | 3 |
| LOIS3 | 465 | 33 | 0.0710 | 12,350 | 12 |
| EOIS3 | 518 | 60 | 0.1158 | 10,150 | 12 |

# 4 Correlations between faunal δ^15^N and bioclimatic variables and covariate selection for geostatistical analyses

The bioclimatic variables from Beyer et al. [28] considered for inclusion as fixed effects in geostatistical analysis are given in Table S4.1. Many of these variables displayed significant correlation with faunal δ^15^N (Table S4.2) and a high degree of collinearity with one another (Fig S4.1). In selecting which variables and combinations of variables to consider as fixed effects in the liner mixed-effects models, a number of aspects were considered. First, we considered variables which in the modern environment have demonstrated empirical relationships with δ^15^N; mean annual temperature (MAT), mean annual precipitation (MAP) and elevation [29–32]. Based on investigated correlations (Table S4.2) we retained MAT and MAP and removed elevation, which showed significant correlation with δ^15^N for only two time bins. While elevation is an important spatial variable across which δ^15^N varies in the modern environment [29,32], there are challenges in estimating palaeo-elevations related to sea-level fluctuations and isostatic responses of the growth and melting of ice sheets. Moreover, our sample set is biased toward environments of <500m elevation, and uncertainties in species-based altitudinal mobility compound uncertainties. Second, we removed variables which provided largely redundant information and displayed high collinearity (Fig S4.1). This included removing precipitation amount of the wettest and driest months, which were highly correlated with precipitation amount of the wettest and driest quarters (correlation coefficients of 0.98 and 0.96, *p* < 0.001); removing minimum and maximum annual temperature which were highly correlated with temperature of the coldest and warmest quarters (correlation coefficients of 0.99 and 0.95, *p* < 0.001); and removing annual temperature range which was highly correlated with temperature seasonality (0.95, *p* < 0.001). Finally, of the remaining variables, those which displayed significant correlations with less than 50% of the time bins were removed. After this, the remaining variables, selected as fixed effects in model testing were: MAT, MAP, temperature of the warmest quarter, precipitation of the warmest quarter and precipitation of the coldest quarter (Fig S4.2 – S4.6).

**Table S4.1. Summary of covariate data considered in this study.**

| **Variable** | **Unit** |
| --- | --- |
| Annual mean temperature | °C |
| Temperature seasonality (standard deviation of monthly temperature) | °C |
| Minimum annual temperature | °C |
| Maximum annual temperature | °C |
| Temperature annual range (difference between minimum and maximum annual temperatures) | °C |
| Mean temperature of the wettest quarter | °C |
| Mean temperature of driest quarter | °C |
| Mean temperature of warmest quarter | °C |
| Mean temperature of coldest quarter | °C |
| Annual precipitation | mm year^-1^ |
| Precipitation of wettest month | mm month^-1^ |
| Precipitation of driest month | mm month^-1^ |
| Precipitation seasonality (coefficient of variation of monthly precipitation) | - |
| Precipitation of wettest quarter | mm quarter^-1^ |
| Precipitation of driest quarter | mm quarter^-1^ |
| Precipitation of warmest quarter | mm quarter^-1^ |
| Precipitation of coldest quarter | mm quarter^-1^ |

**Table S4.2. Pearson’s correlation coefficient indicating correlation between spatial and bioclimatic data and d15N values. Significance indicated by: *****p* < .0001, ****p* < .001, ** *p* < .01, **p* < .05.**

|  | Test statistics | | | | | | | |
| --- | --- | --- | --- | --- | --- | --- | --- | --- |
|  | ALL | EH | YD | LGI | LGT | LGM | LOIS3 | EOIS3 |
| SPATIAL VARIABLES | | | | | | | | |
| Elevation | -0.20**** | -0.21 | -0.37* | -0.19 | -0.27* | -0.07 | 0.12 | -0.05 |
| BIOCLIMATIC VARIABLES | | | | | | | | |
| Annual mean temperature | 0.17*** | 0.18 | 0.12 | 0.71**** | 0.75**** | 0.69**** | 0.27* | 0.17 |
| Temperature seasonality (standard deviation of monthly temperature) | 0.18*** | 0.16 | -0.22 | 0.41*** | -0.16 | -0.27 | -0.1 | -0.07 |
| Minimum annual temperature | 0.09 | 0.09 | 0.34* | 0.47**** | 0.63**** | 0.63**** | 0.16 | 0.14 |
| Maximum annual temperature | 0.25**** | 0.32** | -0.21 | 0.69**** | 0.55**** | 0.60**** | 0.37** | 0.17 |
| Temperature annual range (difference between minimum and maximum annual temperatures) | 0.16*** | 0.16 | -0.28 | 0.37*** | -0.07 | -0.41* | 0 | -0.09 |
| Mean temperature of the wettest quarter | 0.02 | 0.22 | -0.28 | 0.18 | -0.37** | -0.22 | -0.14 | -0.01 |
| Mean temperature of driest quarter | 0.17*** | 0.04 | 0.32 | 0.47**** | 0.64**** | 0.61**** | 0.11 | 0.06 |
| Mean temperature of warmest quarter | 0.29**** | 0.33** | -0.12 | 0.75**** | 0.64**** | 0.69**** | 0.32* | 0.18 |
| Mean temperature of coldest quarter | 0.08 | 0.05 | 0.29 | 0.48**** | 0.65**** | 0.63**** | 0.2 | 0.12 |
| Mean annual precipitation | -0.24**** | -0.41*** | 0.17 | -0.31** | -0.08 | 0.12 | -0.46*** | -0.07 |
| Precipitation of wettest month | -0.19**** | -0.36** | 0.26 | -0.13 | -0.03 | 0.15 | -0.49**** | -0.12 |
| Precipitation of driest month | -0.24**** | -0.50**** | 0.18 | -0.48**** | -0.15 | 0.04 | -0.36** | -0.01 |
| Precipitation seasonality (coefficient of variation of monthly precipitation) | 0.02 | 0.11 | 0.41* | 0.54**** | -0.24* | -0.11 | 0 | -0.12 |
| Precipitation of wettest quarter | -0.19**** | -0.36** | 0.26 | -0.22 | -0.05 | 0.14 | -0.47**** | -0.08 |
| Precipitation of driest quarter | -0.23**** | -0.47**** | 0.1 | -0.39*** | -0.1 | -0.01 | -0.32* | -0.01 |
| Precipitation of warmest quarter | -0.31**** | -0.36** | -0.05 | -0.44**** | -0.48**** | -0.51** | -0.38** | -0.06 |
| Precipitation of coldest quarter | -0.13** | -0.38** | 0.3 | -0.24* | 0.19 | 0.42* | -0.36** | 0 |

**
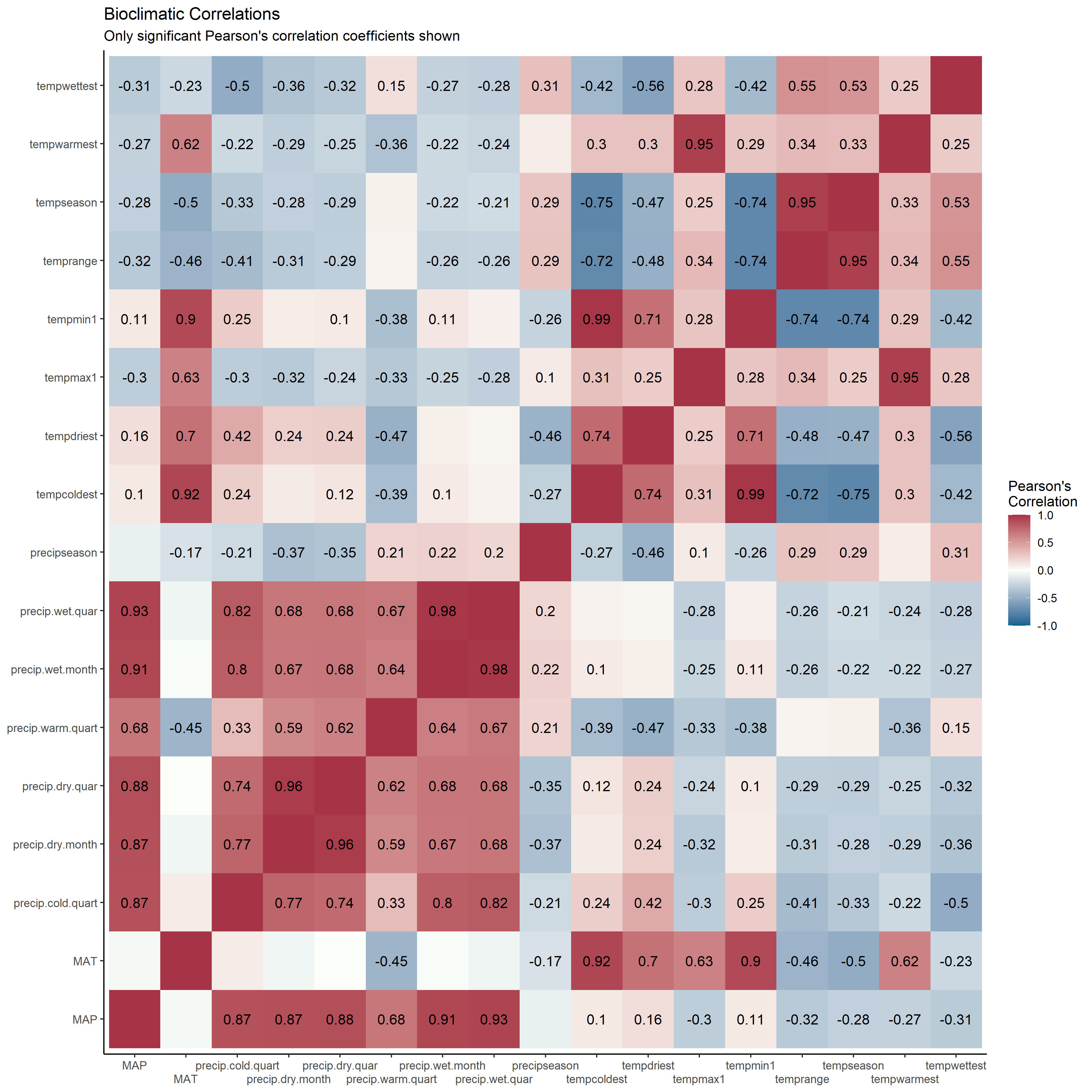
**

**Figure S4.1 Correlation matrix of bioclimatic variables from Beyer et al. (2020). Pearson’s correlation test statistics is displayed only where correlation is significant at p > 0.05.**


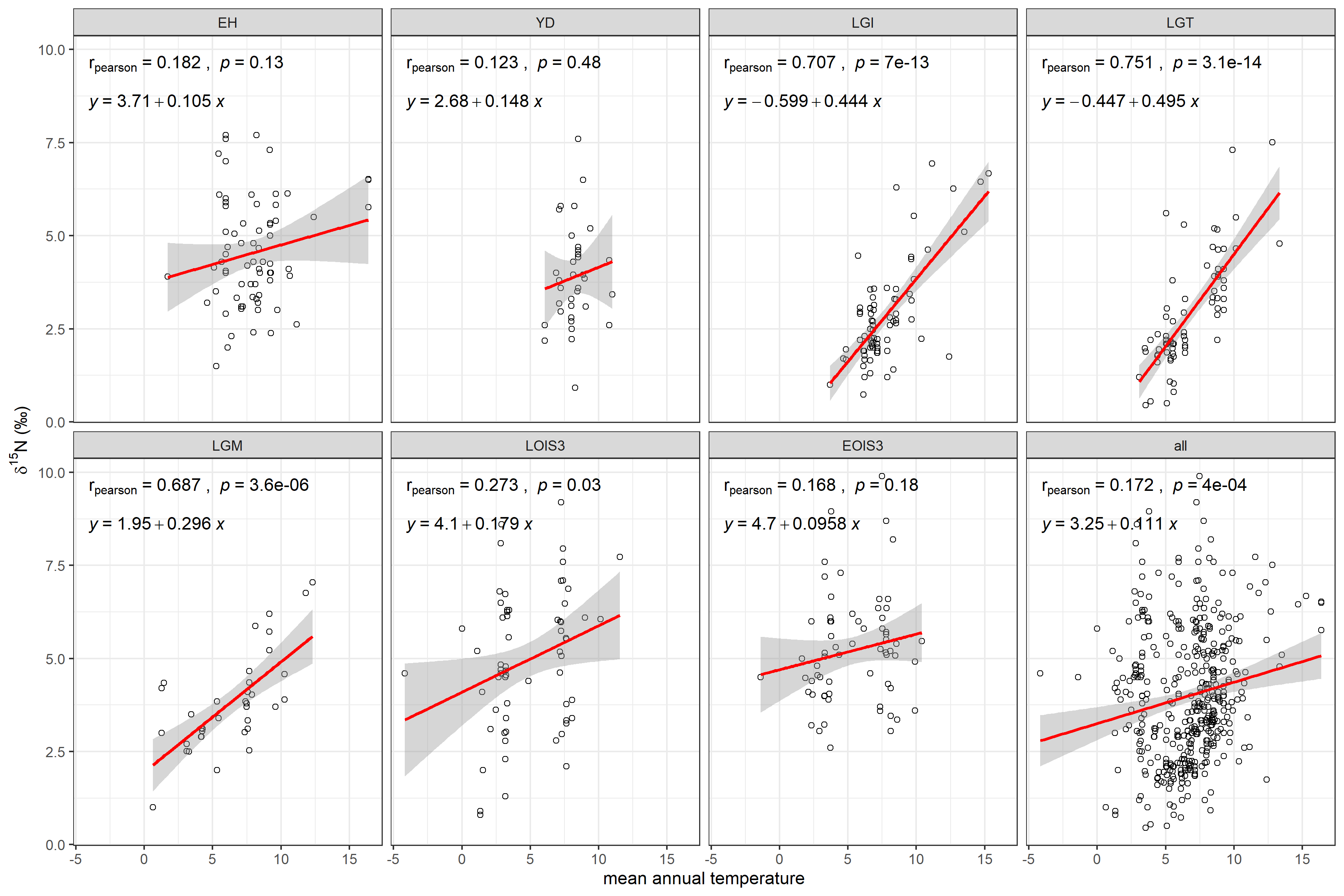
**Figure S4.2 Relationship between site mean faunal δ^15^N and mean annual temperature as derived from the bioclimatic model outputs of Beyer et al. [28].**


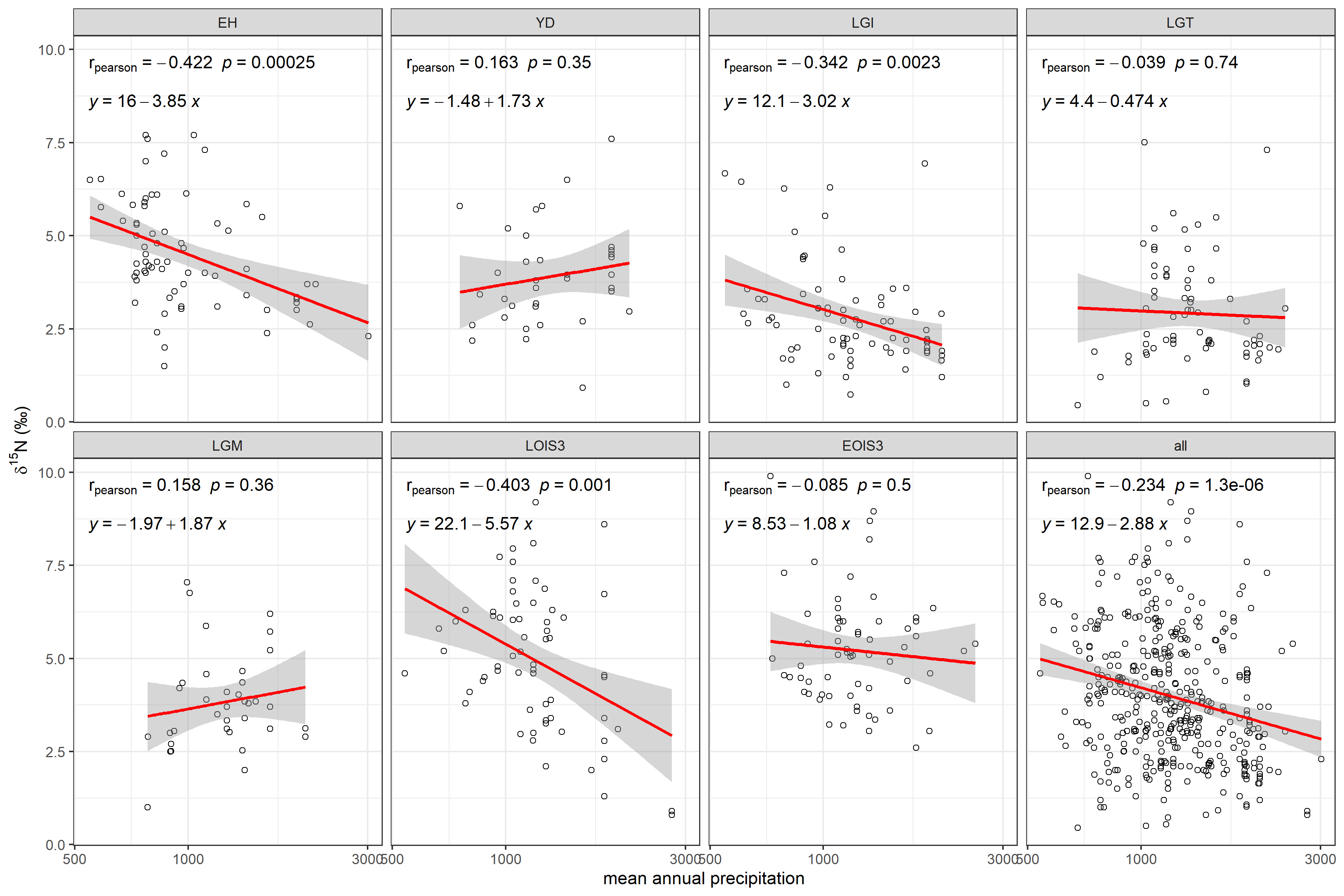
**Figure S4.3 Relationship between site mean faunal δ^15^N and mean annual precipitation as derived from the bioclimatic model outputs of Beyer et al. [28].**


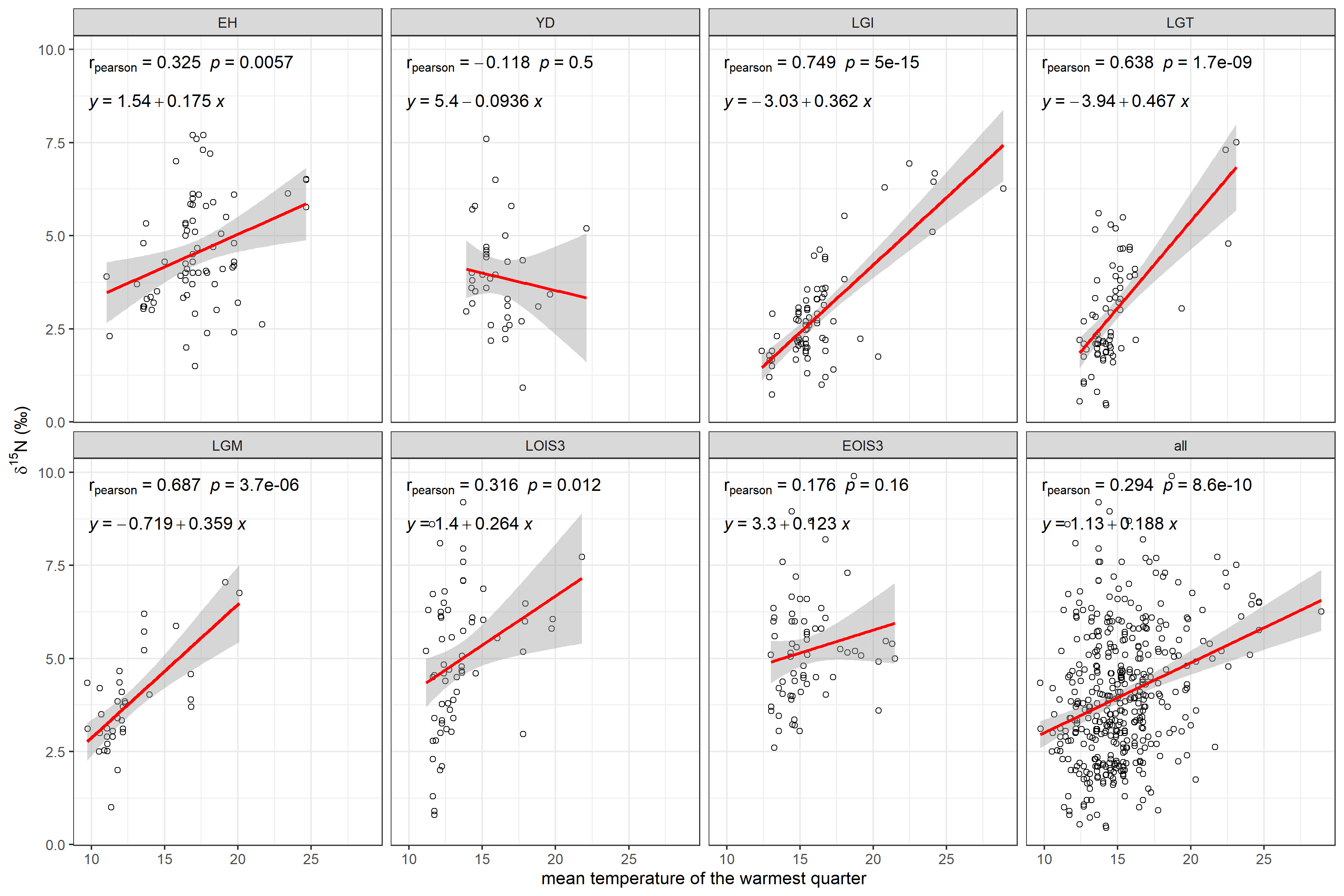
**Figure S4.4 Relationship between site mean faunal δ^15^N and mean temperature of the warmest quarter as derived from the bioclimatic model outputs of Beyer et al. [28].**


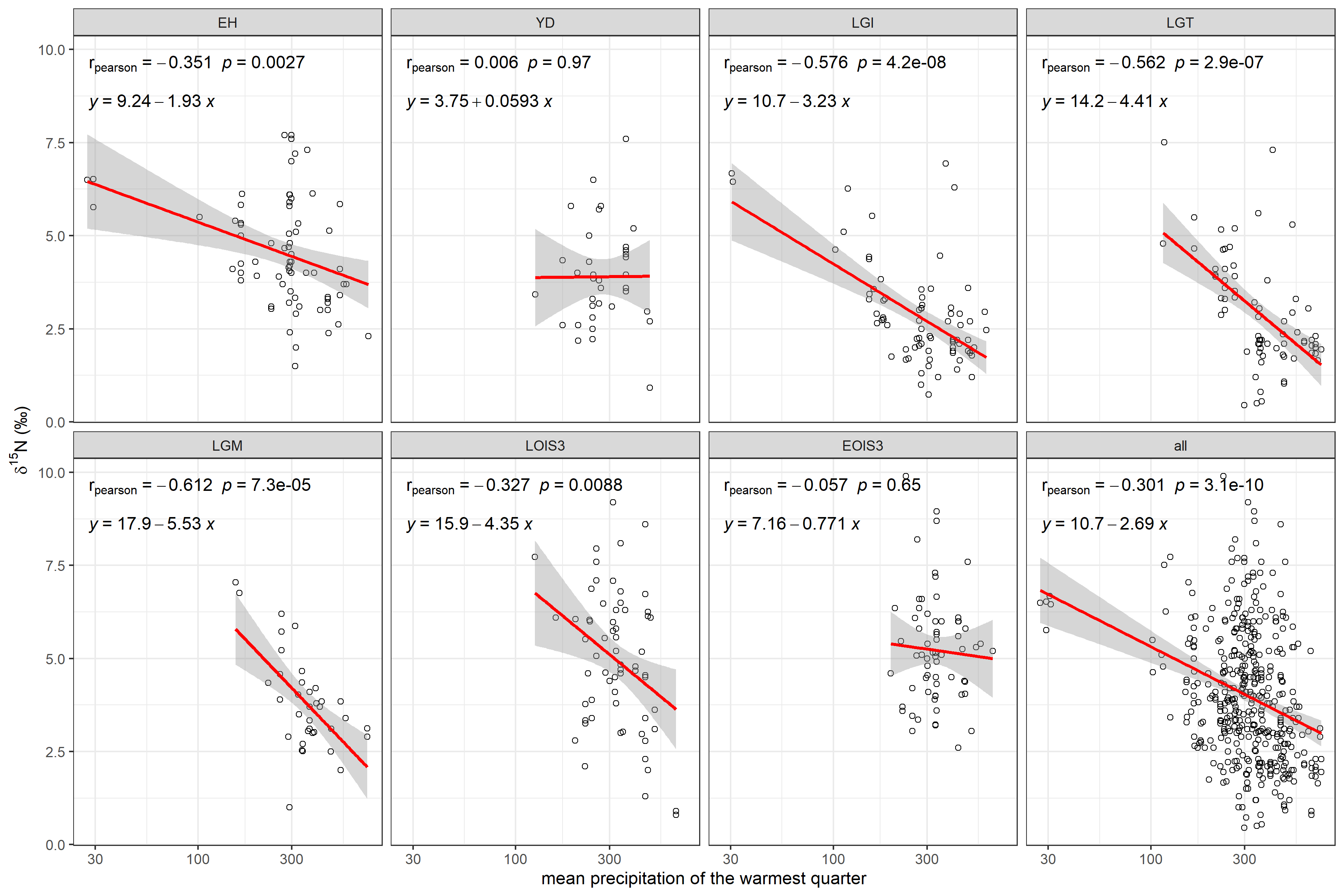
**Figure S4.5 Relationship between site mean faunal δ^15^N and mean precipitation of the warmest quarter as derived from the bioclimatic model outputs of Beyer et al. [28].**


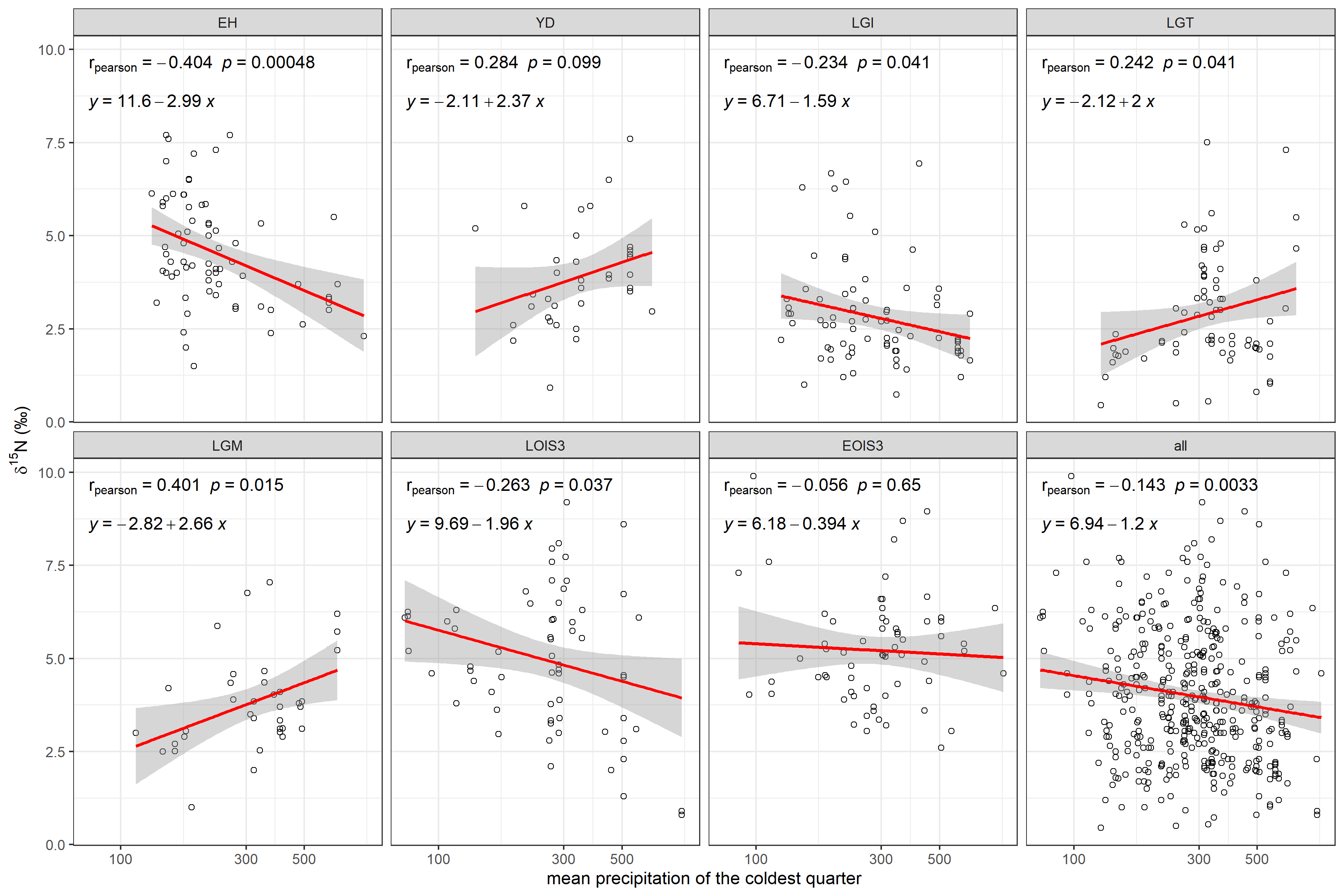
**Figure S4.6 Relationship between site mean faunal δ^15^N and mean precipitation of the coldest quarter as derived from the bioclimatic model outputs of Beyer et al. [28].**

# 5 Isoscape prediction model fitting and performance evaluation

**
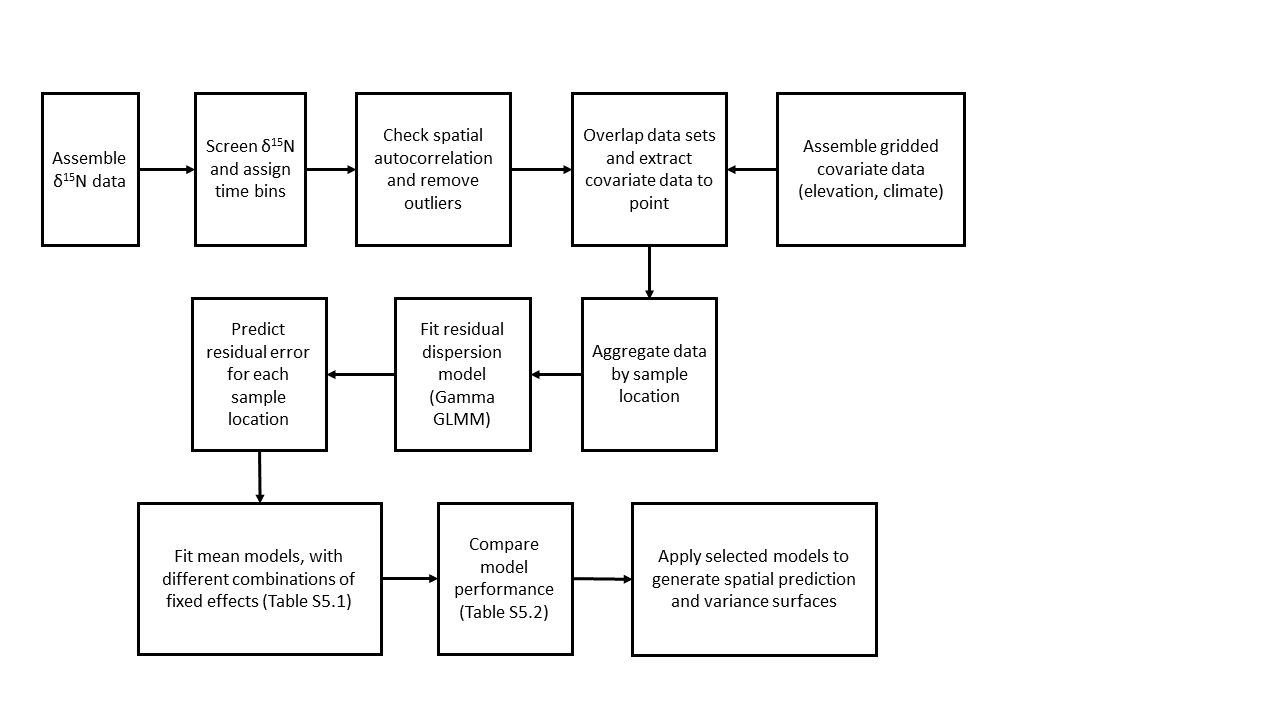
**

**Figure S5.1 Workflow for our analysis described in Section 2 of the main manuscript**

**Table S5.1 Models tested. Each model tested included/excluded a different combination of random effect, fixed effects, and order interaction terms.**

| **Model number** | **Model** |
| --- | --- |
| 1 | Intercept + spatial + uncorrelated |
| 2 | Intercept + MAT + spatial + uncorrelated |
| 3 | Intercept + MAP + spatial + uncorrelated |
| 4 | Intercept + MAT + MAP + spatial + uncorrelated |
| 5 | Intercept + MAT + MAP + MAT:MAP + spatial + uncorrelated |
| 6 | Intercept + temp.warmest.quart + spatial + uncorrelated |
| 7 | Intercept + precip.warmest.quart + spatial + uncorrelated |
| 8 | Intercept + precip.coldest.quart + spatial + uncorrelated |
| 9 | Intercept + temp.warmest.quart + precip.warmest.quart + spatial + uncorrelated |
| 10 | Intercept + temp.warmest.quart + precip.coldest.quart + spatial + uncorrelated |
| 11 | Intercept + precip.coldest.quart + precip.warmest.quart + spatial + uncorrelated |
| 12 | Intercept + temp.warmest.quart + precip.coldest.quart + precip.warmest.quart + spatial + uncorrelated |

**Table S5.2 Model fit results for each model tested (model number corresponds to those given in Table S5.1 and the effects specified therein). The top three performing models, based on cAIC criterion are indicated by ***(best), **(second best), *(third best), and the best performing model’s cAIC, is highlighted in bold red italics.**

|  | **Model number** | **marginal AIC** | **conditional AIC** | **Effective df** |
| --- | --- | --- | --- | --- |
| **Early Holocene** | | | | |
|  | 1 | 259.9 | 213.3 | 26.4 |
|  | 2 | 258.0 | 212.7 | 27.0 |
| * | 3 | 253.1 | 210.9 | 28.3 |
| *** | 4 | 247.0 | ***210.3*** | 29.6 |
|  | 5 | 248.4 | 210.9 | 29.3 |
|  | 6 | 256.0 | 211.9 | 26.6 |
|  | 7 | 251.8 | 211.4 | 28.1 |
|  | 8 | 255.3 | 211.9 | 27.4 |
| ** | 9 | 251.3 | 210.8 | 28.0 |
|  | 10 | 250.9 | 211.2 | 27.2 |
|  | 11 | 250.4 | 211.4 | 28.0 |
|  | 12 | 250.6 | 211.2 | 27.4 |
| **Younger Dryas** | | | | |
| * | 1 | 125.2 | 77.9 | 7.3 |
|  | 2 | 126.9 | 78.0 | 7.4 |
|  | 3 | 126.8 | 77.9 | 7.3 |
|  | 4 | 128.4 | 78.1 | 7.4 |
|  | 5 | 130.5 | 78.3 | 7.1 |
| *** | 6 | 127.9 | ***77.7*** | 7.7 |
|  | 7 | 126.3 | 77.9 | 7.4 |
|  | 8 | 127.5 | 78.0 | 7.2 |
| ** | 9 | 128.3 | 77.8 | 7.6 |
|  | 10 | 129.9 | 78.0 | 7.3 |
|  | 11 | 128.7 | 78.1 | 7.1 |
|  | 12 | 129.1 | 78.0 | 7.4 |
| **Late Glacial Interstadial** | | | | |
| *** | 1 | 198.6 | ***110.5*** | 29.1 |
|  | 2 | 196.6 | 110.8 | 28.2 |
|  | 3 | 200.3 | 110.9 | 28.7 |
|  | 4 | 198.6 | 111.2 | 27.9 |
|  | 5 | 200.7 | 111.6 | 27.7 |
| ** | 6 | 194.6 | 110.7 | 28.8 |
| * | 7 | 199.2 | 110.8 | 28.4 |
|  | 8 | 201.5 | 111.1 | 28.8 |
|  | 9 | 196.6 | 111.1 | 28.3 |
|  | 10 | 196.2 | 111.2 | 28.7 |
|  | 11 | 199.3 | 111.3 | 28.3 |
|  | 12 | 197.9 | 111.4 | 28.2 |
| **Last Glacial Termination** | | | | |
|  | 1 | 227.8 | 132.6 | 22.9 |
| *** | 2 | 201.2 | ***131.8*** | 22.7 |
|  | 3 | 229.1 | 132.9 | 22.8 |
|  | 4 | 201.1 | 132.4 | 22.8 |
|  | 5 | 202.4 | 132.5 | 22.4 |
| ** | 6 | 211.6 | 131.9 | 24.0 |
|  | 7 | 221.6 | 133.1 | 23.4 |
|  | 8 | 227.7 | 132.9 | 22.7 |
|  | 9 | 210.4 | 132.2 | 24.4 |
| * | 10 | 213.7 | 132.1 | 23.7 |
|  | 11 | 218.3 | 133.3 | 23.7 |
|  | 12 | 211.8 | 132.4 | 24.1 |
| **Last Glacial Maximum** | | | | |
|  | 1 | 112.6 | 54.1 | 10.8 |
|  | 2 | 101.5 | 54.2 | 11.8 |
|  | 3 | 114.0 | 54.3 | 10.7 |
|  | 4 | 104.0 | 54.3 | 11.7 |
|  | 5 | 106.2 | 54.8 | 11.2 |
|  | 6 | 97.2 | 56.6 | 12.7 |
| * | 7 | 111.7 | 54.0 | 11.0 |
| ** | 8 | 109.9 | 53.7 | 11.4 |
|  | 9 | 98.4 | 56.4 | 12.3 |
|  | 10 | 96.1 | 55.6 | 14.0 |
| *** | 11 | 104.7 | ***52.9*** | 12.4 |
|  | 12 | 98.3 | 54.9 | 13.9 |
| **Late OIS 3** | | | | |
|  | 1 | 251.8 | 170.2 | 16.0 |
|  | 2 | 246.5 | 170.8 | 16.5 |
| *** | 3 | 243.9 | ***169.3*** | 16.4 |
| ** | 4 | 241.5 | 169.3 | 17.0 |
|  | 5 | 243.4 | 169.7 | 16.8 |
|  | 6 | 244.7 | 170.4 | 17.0 |
| * | 7 | 243.7 | 169.4 | 16.8 |
|  | 8 | 247.7 | 169.9 | 16.0 |
|  | 9 | 243.5 | 169.8 | 17.0 |
|  | 10 | 245.0 | 170.0 | 16.7 |
|  | 11 | 244.6 | 169.5 | 16.6 |
|  | 12 | 244.7 | 169.8 | 16.7 |
| **Early OIS 3** | | | | |
| * | 1 | 226.3 | 181.2 | 35.1 |
| *** | 2 | 227.2 | ***181.0*** | 34.4 |
|  | 3 | 228.4 | 181.9 | 34.4 |
| ** | 4 | 229.2 | 181.1 | 33.5 |
|  | 5 | 231.1 | 182.0 | 32.9 |
|  | 6 | 228.1 | 181.8 | 34.2 |
|  | 7 | 227.8 | 181.6 | 35.0 |
|  | 8 | 228.3 | 182.2 | 34.8 |
|  | 9 | 229.8 | 182.2 | 34.2 |
|  | 10 | 230.1 | 182.7 | 34.0 |
|  | 11 | 229.9 | 182.5 | 34.9 |
|  | 12 | 232.1 | 183.0 | 34.0 |

**
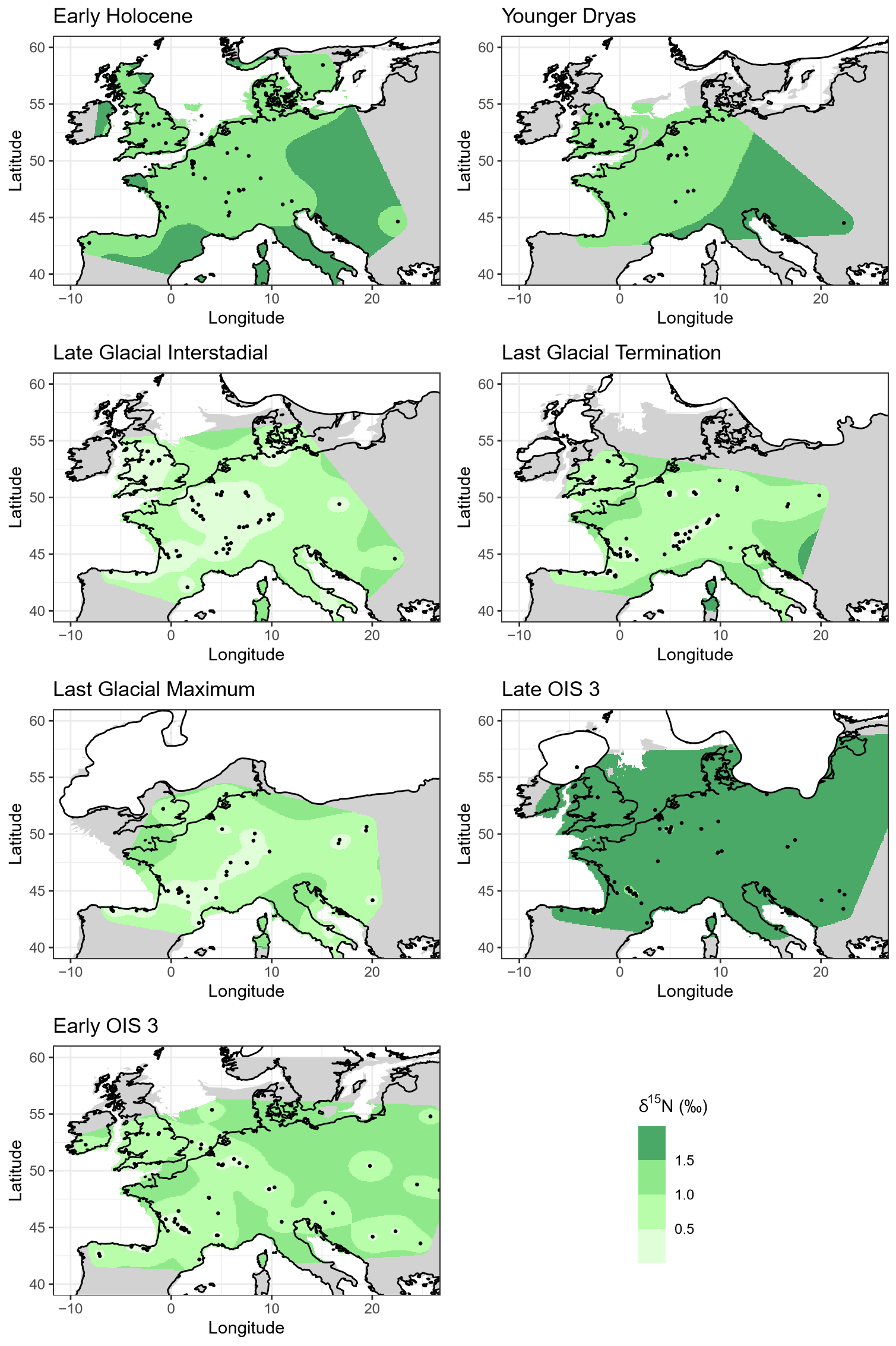
**

**Figure S5.2. δ^15^N isoscape variance surfaces, modelled using random effects only. Palaeocoastline data from** [20] **ice sheet extent from** [21] **and modern coastline from** [22]**.**

**
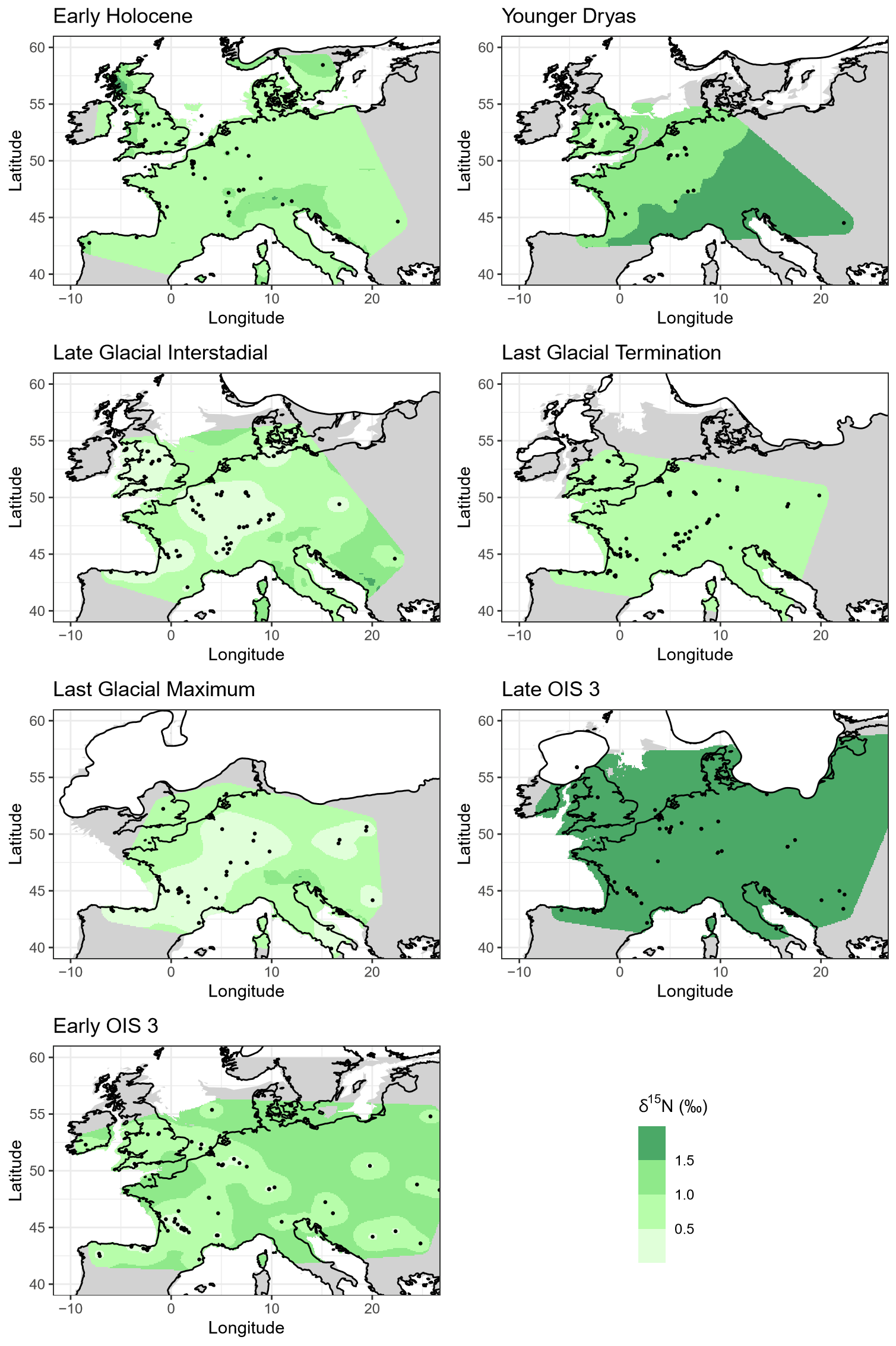
**

**Figure S5.3. δ^15^N isoscape variance surfaces, best performing model incorporating climatic fixed effect(s) for each time bin. Palaeocoastline data from** [20] **ice sheet extent from** [21] **and modern coastline from** [22]**.**


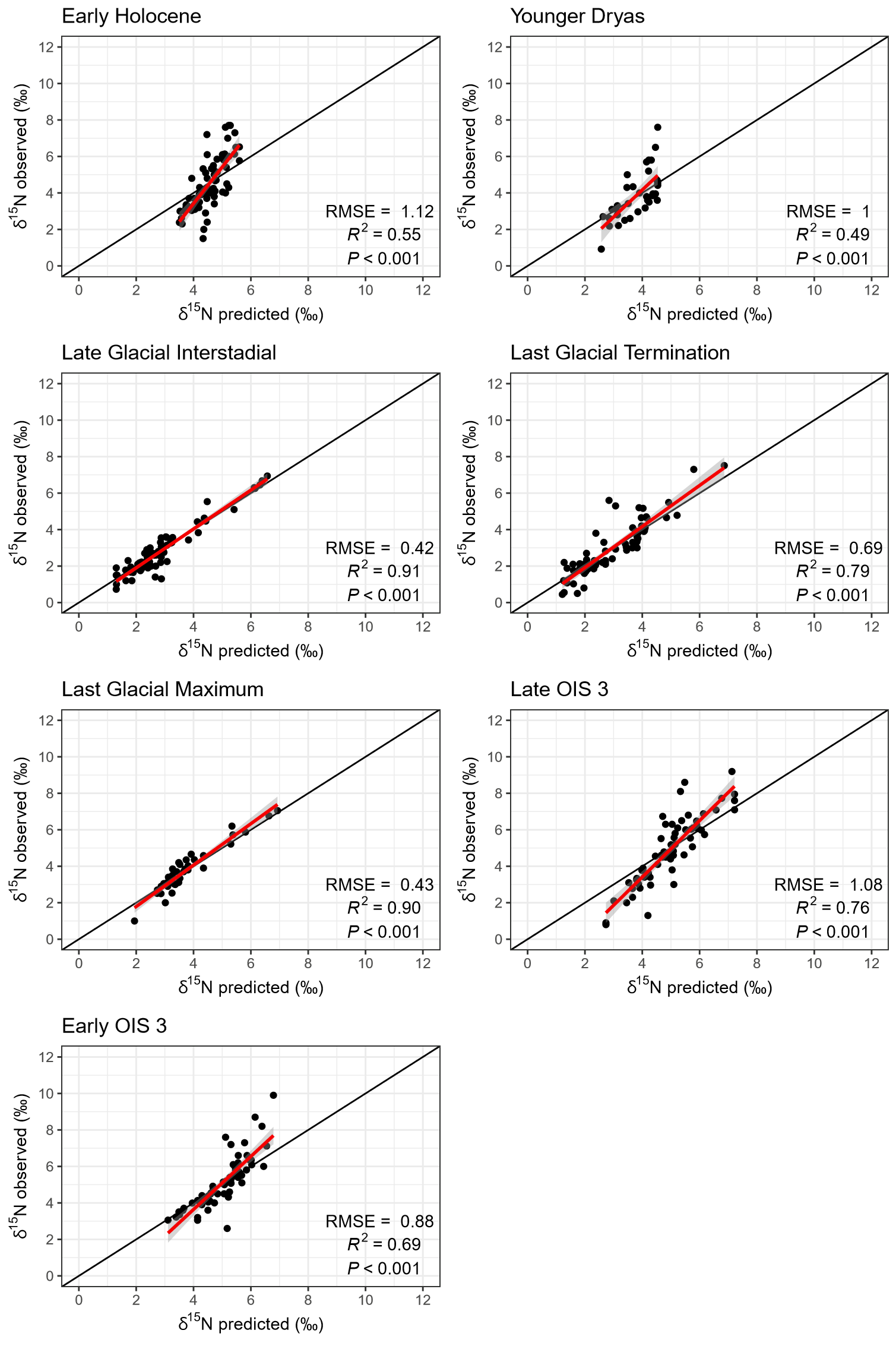


**Figure S5.4 Comparison of observed site mean δ^15^N versus model predicted δ^15^N, for the models using random effects only.**

**
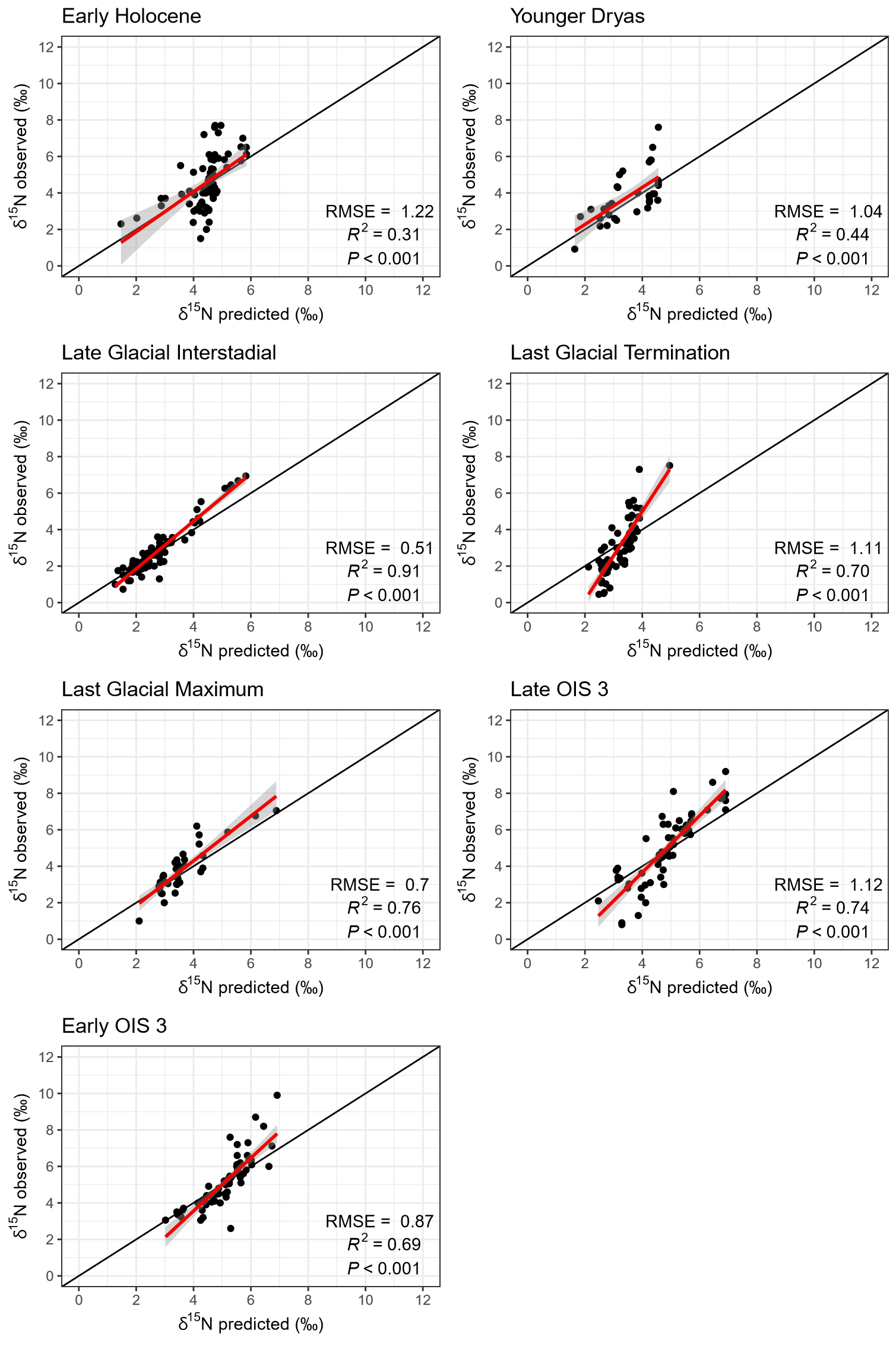
**

Figure S5.**5** Comparison of observed site mean δ^15^N versus model predicted δ^15^N, best performing model incorporating climatic fixed effect(s) for each time bin.

# 6 Species-specific sample distribution


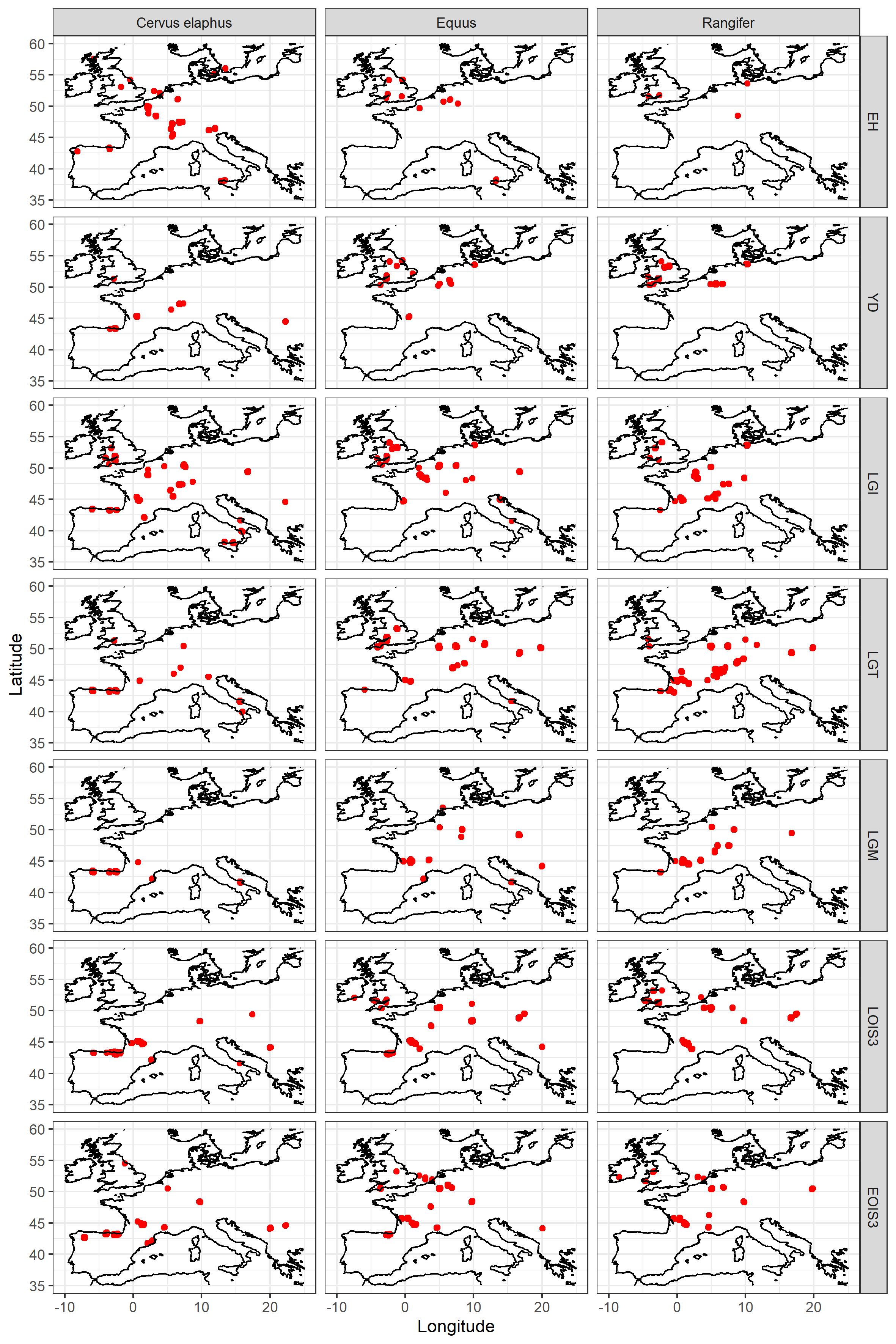


**Figure S6.1 Distribution of *Cervus elaphus, Equus* sp., and *Rangifer tarandus* samples in the compiled data set for each time bin. Modern coastline from** [22]**.**

# 7 Supplementary References

1. Stevens R. Establishing links between climate/environment & both modern & archaeological hair & bone isotope values: Determining the potential of archaeological bone collagen d13C and d15N as palaeoclimatic and palaeoenvironmental proxies. University of Oxford; DPhil thesis. 2005.

2. Brock F, Higham T, Ditchfield P, Ramsey CB. Current Pretreatment Methods for AMS Radiocarbon Dating at the Oxford Radiocarbon Accelerator Unit (ORAU). Radiocarbon. 2010 Jul 18;52(1):103–12. doi: 10.1017/S0033822200045069

3. Longin R. New Method of Collagen Extraction for Radiocarbon Dating. Nature. 1971 Mar;230(5291):241–2. doi: 10.1038/230241a0

4. Stevens RE, Hedges REM. Carbon and nitrogen stable isotope analysis of northwest European horse bone and tooth collagen, 40,000 BP-present: Palaeoclimatic interpretations. Quat Sci Rev. 2004. p. 977–91. doi: 10.1016/j.quascirev.2003.06.024

5. Stevens RE, Hermoso-Buxán XL, Marín-Arroyo AB, González-Morales MR, Straus LG. Investigation of Late Pleistocene and Early Holocene palaeoenvironmental change at El Mirón cave (Cantabria, Spain): Insights from carbon and nitrogen isotope analyses of red deer. Palaeogeogr Palaeoclimatol Palaeoecol. 2014 Jun 20;414:46–60. doi: 10.1016/j.palaeo.2014.05.049

6. Sayle KL, Brodie CR, Cook GT, Hamilton WD. Sequential measurement of δ ^15^ N, δ ^13^ C and δ ^34^ S values in archaeological bone collagen at the Scottish Universities Environmental Research Centre (SUERC): A new analytical frontier. Rapid Commun Mass Spectrom. 2019 Aug 15;33(15):1258–66. doi: 10.1002/rcm.8462

7. Stevens RE, Jacobi R, Street M, Germonpré M, Conard NJ, Münzel SC, et al. Nitrogen isotope analyses of reindeer (Rangifer tarandus), 45,000 BP to 9,000 BP: Palaeoenvironmental reconstructions. Palaeogeogr Palaeoclimatol Palaeoecol. 2008 May 27;262(1–2):32–45. doi: 10.1016/j.palaeo.2008.01.019

8. Bronk Ramsey C, Humm MJ. On-line combustion of samples for AMS and ion source developments at ORAU. Nuclear Instruments and Methods in Physics Research Section B: Beam Interactions with Materials and Atoms. 2000 Oct;172(1–4):242–6.

9. Dee M, Bronk Ramsey C. Refinement of graphite target production at ORAU. Nucl Instrum Methods Phys Res B. 2000 Oct;172(1–4):449–53. doi: 10.1016/S0168-583X(00)00337-2

10. Bronk Ramsey C, Hedges REM. Hybrid ion sources: Radiocarbon measurements from microgram to milligram. Nucl Instrum Methods Phys Res B. 1997 Mar;123(1–4):539–45. doi:10.1016/S0168-583X(96)00612-X

11. Bronk Ramsey C, Higham T, Leach P. Towards High-Precision AMS: Progress and Limitations. Radiocarbon. 2004 Jul 18;46(1):17–24. doi:10.1017/S0033822200039308

12. Wood RE, Ramsey CB, Higham TFG. Refining Background Corrections for Radiocarbon Dating of Bone Collagen at Orau. Radiocarbon. 2010 Jul 18;52(2):600–11. doi:10.1017/S003382220004563X

13. Reade H, Tripp JA, Charlton S, Grimm S, Sayle KL, Fensome A, et al. Radiocarbon chronology and environmental context of Last Glacial Maximum human occupation in Switzerland. Sci Rep. 2020 Dec 13;10(1):4694. doi: 10.1038/s41598-020-61448-7

14. Bronk Ramsey C. Bayesian Analysis of Radiocarbon Dates. Radiocarbon. 2009 Jul 18;51(1):337–60. doi: 10.1017/S0033822200033865

15. Reimer PJ, Austin WEN, Bard E, Bayliss A, Blackwell PG, Bronk Ramsey C, et al. The IntCal20 Northern Hemisphere Radiocarbon Age Calibration Curve (0–55 cal kBP). Radiocarbon. 2020 Aug 12;62(4):725–57. doi: doi:10.1017/RDC.2020.41

16. Hofman‐Kamińska E, Bocherens H, Drucker DG, Fyfe RM, Gumiński W, Makowiecki D, et al. Adapt or die—Response of large herbivores to environmental changes in Europe during the Holocene. Glob Chang Biol. 2019 Sep 12;25(9):2915–30. doi: 10.1111/gcb.14733

17. Schwartz-Narbonne R, Longstaffe FJ, Kardynal KJ, Druckenmiller P, Hobson KA, Jass CN, et al. Reframing the mammoth steppe: Insights from analysis of isotopic niches. Quat Sci Rev. 2019; 215:1–21. doi: 10.1016/j.quascirev.2019.04.025

18. Drucker DG. The Isotopic Ecology of the Mammoth Steppe. Annu Rev Earth Planet Sci. 2022 May 30;50(1):395–418. doi: 10.1146/annurev-earth-100821-081832

19. Szpak P. Complexities of nitrogen isotope biogeochemistry in plant-soil systems: implications for the study of ancient agricultural and animal management practices. Front Plant Sci. 2014 Jun 23;5(JUN):288. doi: 10.3389/fpls.2014.00288

20. Zickel M, Becker D, Verheul J, Yener Y, Willmes C. Paleocoastlines GIS dataset; computed land masks of past sea level models. CRC806-Database [Internet]. 2016. Available from: https://crc806db.uni-koeln.de/dataset/show/paleocoastlines-gis-dataset1462293239/ doi: 10.5880/SFB806.20 (CC BY 4.0 license)

21. Hughes ALC, Gyllencreutz R, Lohne OS, Mangerud J, Svendsen JI DATED-1: compilation of dates and time-slice reconstruction of the build-up and retreat of the last Eurasian (British-Irish, Scandinavian, Svalbard-Barents-Kara Seas) Ice Sheets 40-10 ka. 2015. Department of Earth Science, University of Bergen and Bjerknes Centre for Climate Research, PANGAEA. Available from: https://doi.org/10.1594/PANGAEA.848117 (CC BY 3.0 license)

22. Natural Earth. 1:10m Coastline [Internet]. Free vector and raster map data. [cited 2022 Nov 4]. Available from: https://www.naturalearthdata.com/

23. Hansen RM. Foods of Free-Roaming Horses in Southern New Mexico. Journal of Range Management. 1976 Jul;29(4):347.

24. Ben-David M, Shochat E, Adams LG. Utility of stable isotope analysis in studying foraging ecology of herbivores: Examples from moose and caribou. Alces [Internet]. 2001;37(2):421–34.

25. Rivals F, Semprebon GM. Latitude matters: an examination of behavioural plasticity in dietary traits amongst extant and Pleistocene Rangifer tarandus. Boreas. 2017 Apr 1;46(2):254–63. doi: 10.1111/bor.12205

26. Drucker DG, Hobson KA, Ouellet JP, Courtois R. Influence of forage preferences and habitat use on ^13^ C and ^15^ N abundance in wild caribou ( *Rangifer tarandus caribou* ) and moose ( *Alces alces* ) from Canada. Isotopes Environ Health Stud [Internet]. 2010 Mar;46(1):107–21. doi: 10.1080/10256010903388410

27. Tahmasebi F, Longstaffe FJ, Zazula G, Bennett B. Nitrogen and carbon isotopic dynamics of subarctic soils and plants in southern Yukon Territory and its implications for paleoecological and paleodietary studies. Mohan J, editor. PLOS ONE. 2017 Aug 16;12(8):e0183016. doi: 10.1371/journal.pone.0183016

28. Beyer RM, Krapp M, Manica A. High-resolution terrestrial climate, bioclimate and vegetation for the last 120,000 years. Sci Data. 2020 Dec 1;7(1). doi: 10.1038/s41597-020-0552-1

29. Männel TT, Auerswald K, Schnyder H. Altitudinal gradients of grassland carbon and nitrogen isotope composition are recorded in the hair of grazers. Glob Ecol Biogeogr. 2007 Sep;16(5):583–92. doi: 10.1111/j.1466-8238.2007.00322.x

30. Craine JM, Elmore AJ, Aidar MPM, Bustamante M, Dawson TE, Hobbie EA, et al. Global patterns of foliar nitrogen isotopes and their relationships with climate, mycorrhizal fungi, foliar nutrient concentrations, and nitrogen availability. New Phytol. 2009;183:980–92. doi: 10.1111/j.1469-8137.2009.02917.x

31. Craine JM, Brookshire ENJ, Cramer MD, Hasselquist NJ, Koba K, Marin-Spiotta E, et al. Ecological interpretations of nitrogen isotope ratios of terrestrial plants and soils. Plant Soil. 2015;396:1–26. doi: 10.1007/s11104-015-2542-1

32. Liu X, Wang G, Li J, Wang Q. Nitrogen isotope composition characteristics of modern plants and their variations along an altitudinal gradient in Dongling Mountain in Beijing. Sci China Ser D Earth Sci . 2010 Jan 3;53(1):128–40. doi: 10.1007/s11430-009-0175-z
