# Supplementary material for: Nitrogen palaeo-isoscapes: Changing spatial gradients of faunal δ15N in late Pleistocene and early Holocene Europe
Source: PLoS One. 2023 Feb 6;18(2):e0268607. doi: 10.1371/journal.pone.0268607 (PMC9901814; doi:10.1371/journal.pone.0268607)
Supplement: S2 File — (ZIP) [file pone.0268607.s004.zip › S4 Base maps for figures/ne_10m_coastline.README.html]

Natural Earth » Blog Archive » Coastline - Free vector and raster map data at 1:10m, 1:50m, and 1:110m scales 


# 

Free vector and raster map data at 1:10m, 1:50m, and 1:110m scales

Search for:

- Home
- Features
- Downloads
- Blog
- Issues
- Corrections
- About

---

## Coastline

*Includes major islands.* 

Download coastline (2.93 MB) version 4.1.0

**About**

Ocean coastline, including major islands. Coastline is matched to land and water polygons. The Caspian Sea, which is technically a lake, is included. The ocean coastline, the foundation for building all of NEV, primarily derives from World Data Bank 2 with modest generalization applied via line simplification in Adobe Illustrator. The Antarctica coast derives from NASA Mosaic of Antarctica.

*(below) Yucatan peninsula, Cuba, and Hispaniola.*

**Issues**

World Data Bank 2 coastlines have suspect accuracy for certain parts of the world, including northern Russia, southern Chile, and most egregiously, the west coast of the United States extending to the Baja Peninsula, Mexico, where the coast was approximately 7 km east of where it should be. The west coast of the US was corrected on NEV; other areas were not.

Does not include rank 6, 7, or 8 coastlines from the minor islands. Some rank 5 coastline should be reclassified as rank 6.

**Resources**

- World Data Bank 2
- Mosaic of Antarctica

**Version History**

- 4.1.0
- 4.0.0
- 3.0.0
- 2.0.0
- 1.3.0
- 1.1.0
- 1.0.0

The master changelog is available on Github »

This entry was posted
on Tuesday, November 17th, 2009 at 12:01 am and is filed under 10m-physical-vectors.
You can follow any responses to this entry through the RSS 2.0 feed.
Both comments and pings are currently closed.


Comments are closed.

- Subscribe: Entries | Comments
- ## Search

  Search for:
- ## Links

  - NACIS
- ## Tags

  10m
  50m
  90
  180
  admin-0
  bjorn
  bounding box
  browser
  change log
  corrections
  countries
  Downloads
  error
  extent
  ext js
  forums
  geoext
  hans
  imagery
  import
  mapnik
  maptiler
  map tiles
  marine boundary
  national parks
  new data
  nsd
  openlayers
  physical labels
  pngng
  populated places
  raster
  terrestrial hypsography
  tfw
  thematic mapping
  themese
  tif
  tilecache
  tiles
  time zones
  towns
  transportation
  update
  visitors
  world file
- ## Recent Comments

  - stromectol cvs on Download URLs – double slash
  - stromectol tablets for humans on Download URLs – double slash
  - buy stromectol 6 mg tablets on Download URLs – double slash
  - ivermectin 12 mg otc on Download URLs – double slash
  - The genomic origins of the Bronze Age Tarim Basin mummies – Nature.com – Daily Research on 1:10m Gray Earth
- ## Recent Forum Topics

  - Natural Earth in Wagner VII
    by Hugo Ahlenius
  - Downloads are 404ing
    by Nathaniel
  - Disputed Territories: "type" field
    by alykat
  - ISO code confusion
    by nth
  - Bad ADM1NAME, encoding in version 3.0.0 and missing diacritics in NAME
    by pfunes
  - U.S. County Shape File
    by gzingsheim
  - Projection / Proportion / Compatibility?
    by Liquidized
  - Download URLs – double slash
    by vastur
  - map soft – writer: me
    by krzysztof
  - Unicode encoding issue – ne\_10m\_lakes.dbf
    by filter.1

---

Supported by:

© 2009 - 2021. Natural Earth. All rights reserved.


Powered by WordPress

Staff Login »
